# Recent Advancements in the Development of HDAC/Tubulin Dual-Targeting Inhibitors

**DOI:** 10.3390/ph18030341

**Published:** 2025-02-27

**Authors:** Christine Tran, Abdallah Hamze

**Affiliations:** BioCIS, CNRS (Centre National de Recherche Scientifique), Université Paris-Saclay, 91400 Orsay, France

**Keywords:** histone deacetylase, tubulin, dual compounds

## Abstract

Histone deacetylases (HDACs) have become one of the main targets in cancer therapy due to their involvement in various biological processes, including gene regulation, cell proliferation, and differentiation. Microtubules, as key elements of the cell cytoskeleton, also represent important therapeutic targets in anticancer drugs research. These proteins are involved in diverse cellular functions, especially mitosis, cell signaling, and intracellular trafficking. With the emergence of multi-target therapy during the last decades, the combination of HDAC and tubulin inhibitors has been envisioned as a practical approach for optimizing the therapeutic efficacy of antitumor molecules. HDAC/tubulin dual-targeting inhibitors offer the advantages of the synergistic action of both compounds, along with a significant decrease in their respective toxicities and drug resistance. This review will detail the major recent advancements in the development of HDAC/tubulin dual inhibitors over the last decade and their impact on anticancer drugs discovery.

## 1. Introduction

Cancer is one of the leading causes of death worldwide, with 19.3 million new cases and around 10 million deaths reported in 2020 [1]. This complex disease involves various biological factors, such as epigenetic alterations, which can modulate gene expression. Among these, the significant variation in the reversible acetylation−deacetylation of histones represents a key hallmark of carcinogenesis [2].

**Figure 1:**
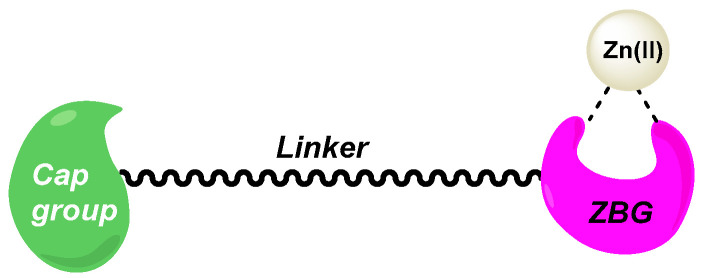
Designed structure of HDACis.

Histone deacetylases (HDACs) are a class of epigenetic metalloenzymes capable of removing acetyl groups from *N*-acetylated lysine residues on histone and non-histone proteins. HDACs mainly target histones to tightly wrap DNA. They can regulate gene expression and protein activity by varying DNA accessibility [3]. In contrast, their dysregulation can lead to the alteration of oncogenes or tumor suppressor gene transcription, inducing carcinogenic events [4]. Hence, HDAC inhibitors (HDACis) have been developed during the last decades as major molecules for cancer treatment [5]. These compounds can inhibit the proliferation and differentiation of cancer cells and promote apoptosis. Their structures are established according to the X-ray crystallographic data of various HDAC isoforms [6,7], and consist of three main features to display effective inhibition: (1) a zinc-binding group (ZBG) chelating the Zn(II) ion in the active site, (2) a linker motif filling the hydrophobic tubular pocket, and (3) a cap unit interacting with the amino acids localized at the edge of the tube-like pocket (Figure 1).

**Figure 2:**
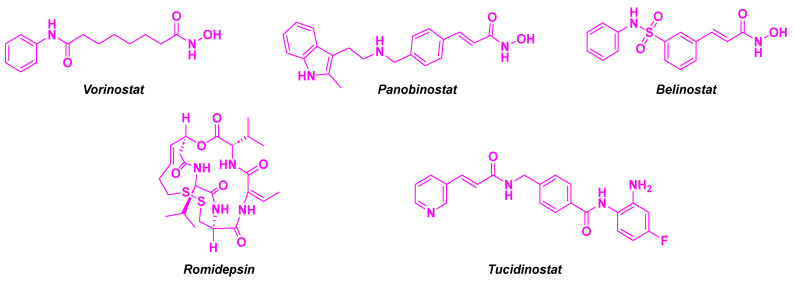
Approved HDACis.

HDACis are promising antitumor agents, with four US FDA (Food and Drug Administration)-approved HDACis (vorinostat, Panobinostat, Belinostat and Romidepsin) [8]. Clinical trials have confirmed their high efficiency in treating solid and liquid tumors [9]. Recently, Tucinidostat was approved as an HDACi in China for relapsed and refractory peripheral T-cell lymphoma [10] (Figure 2). Nonetheless, the main drawback of these HDACis is their low HDAC isoform specificity, causing significant side effects, e. g. fatigue, nausea, thrombocytopenia, cardiotoxicity, or hematologic toxicity [11]. Furthermore, drug resistance to HDACis could also be noted, probably due to the activation of signal transduction pathways, namely Akt (protein kinase B) or CDK (cyclin-dependent protein kinase) signaling processes [12]. Thus, targeting specific HDAC isoforms remains a challenge in the development of novel anticancer therapies [13]. In fact, the overexpression of various types of HDACs could be correlated to a particular type of solid or hematologic cancer. For instance, the high transcription of HDACs 8 and 10 indicates an advanced state of neuroblastoma [14,15]. High levels of HDACs 2, 5, and 9 are present in medulloblastoma, which is associated with poor prognosis [16,17]. Lung cancer cells exhibit aberrant expression of HDACs 1, 2, 3, 5, and 10 [18,19,20]. The unusual regulation of HDACs 1, 2, 3, 4, and 10 is a hallmark of gastric cancers [21,22,23]. Liver cancer cell lines develop increased levels of HDACs 1, 2, 3, and 5 [24,25,26,27,28], and a downregulation of HDAC 6 [29]. The generation of human pancreatic cancer cells is influenced by the concomitant increase in HDACs 2, 6, and 7 [30,31,32]. HDACs 1, 2, 3, 5, and 7 are highly expressed in colorectal cancer [33,34]. The upregulation of HDACs 1, 2, 3, and 6 has been noted in breast cancer [35,36]. Cervical cancer is characterized by a low concentration of HDAC 10 [37]. HDACs 1, 2, and 3 are overexpressed in ovarian [38], prostate [39], and bladder cancers [40]. Concerning hemotological tumors, some are less sensitive to HDAC isoforms, compared to solid ones. A high rate of HDACs 1–9 has been observed in acute lymphocytic leukemia [41]. Analogously, HDACs 1, 3, 6, 7, 9, and 10 are overexpressed in advanced-stage chronic lymphocytic leukemia [42]. Diffuse large B-cell lymphoma has high HDACs 1, 2, 3, and 6 levels [43,44,45,46]. In contrast, acute myeloid leukemia is more specifically defined by a high quantity of HDAC 6 and a low rate of HDAC 5 [47]. Highly expressed HDAC 2 is a prognosis biomarker for aggressive cutaneous T-cell lymphoma [48]. Likewise, HDACs 1, 2, and 3 are upregulated in Hodgkin’s lymphoma cells [49]. The high expression of HDAC 1 could also be a marker of poor prognosis in myeloma [50].

To circumvent all these issues, research efforts on dual-targeting HDACis have emerged. This strategy involves the association of different pharmacophores in a single drug, which can interact with multiple cancer targets. These hybrid molecules present several advantages over drug combinations, such as more predictable pharmacodynamic and pharmacokinetic properties, lower toxicity, and higher efficiency in advanced-stage diseases owing to their synergistic effects [51,52]. Several promising biological targets have been selected as complementary inhibitors of HDACis [12]. As main examples, dual PTK (Protein Kinase) and HDAC inhibitors have been widely developed, namely EGFR (Epidermal Growth Factor Receptor)-HDAC hybrid inhibitors efficient in liver, pancreatic, breast, and lung human cancer cell lines [53], or hybrid BCR/ABL-HDAC inhibitors displaying promising cytotoxicities against various leukemic cell lines [54]. Given the strong implication of JAK (Janus Kinase) in the STAT (Signal Transducers and Activators of Transcription) proteins pathways in cell functions, JAK-HDAC dual inhibitory agents have also been validated against solid and hematologic cancer cells [55]. CDK (Cyclin-Dependent Kinases) inhibitors can also be coupled with HDACi, affording dual hybrids with nanomolar activities against HDAC1 and CDK2 [56]. Given the major involvement of PI3K (Phosphatidylinositol 3-Kinase) pathway alterations in cancer processes [57], dual inhibitors targeting PI3K and HDAC were developed. These hybrids exhibited significant antiproliferative activities in diverse cancer cell lines and favorable pharmacokinetic parameters in vivo [58]. As topoisomerase inhibitors were widely used in cancer therapy [59], their combination with HDACi paved the way for potent dual hybrids impeding cancer cell proliferation [60]. The key role of NAMPT (NicotinAMide PhosphoribosylTransferase) in the synthesis of NAD^+^, which regulates the cell redox process, led to the conception of dual NAMPT/HDAC inhibitors. These molecules aimed to hamper cancer cell growth, by diminishing the concentration of NAD^+^ and favoring apoptosis [61]. Nanomolar values were found for the inhibitory activities toward NAMPT and HDAC 1 for such compounds [61]. The analogous properties of the BET (Bromodomain and Extra Terminal) family and HDACs, such as their action on epigenetic mechanisms, allow access to dual hybrids with subnanomolar cytotoxic and enzymatic activities [62]. The association between PARP (poly (ADP-ribose) polymerase) inhibitors and HDACi was also envisioned, with significant outcomes against cisplatin-resistant SGC7901 cells [63]. Hsp90 (Heat shock protein 90), which participates in the folding of oncoproteins, is a consistent co-target with HDAC in the creation of dual hybrids [64]. In fact, their close interactions in biological pathways, namely between HDAC 6 and Hsp 90 [65], led to the development of dual Hsp90/HDAC inhibitors, with nanomolar inhibitory activities for the corresponding key targets [66].

Among the principal advances made in the field of HDACis with dual-targeting properties, we will particularly focus on dual inhibitors targeting HDACs and tubulin as potential anticancer agents.

**Figure 3:**
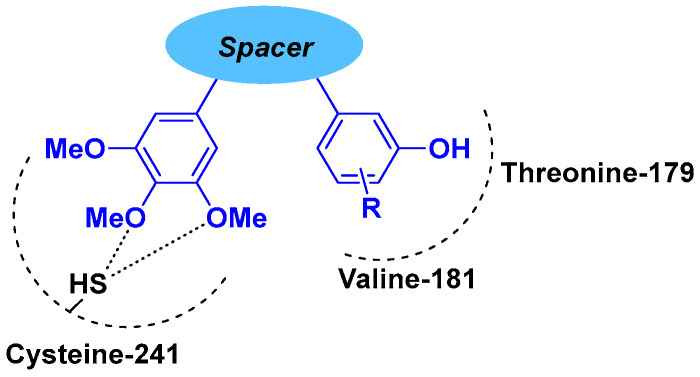
Designed structure of CSBIs.

Microtubules are also a major therapeutic target in the development of new anticancer drugs. They are composed of dynamic cytoskeletal proteins made of α/β-tubulin heterodimers. These structural cell elements play pivotal roles in the maintenance of cell architecture, migration, and proliferation in eukaryotic cells [67]. Tubulin inhibitors, via their interaction with binding sites (laulimalide, taxane/epothilone, vinca alkaloid, and colchicine sites), would stop the cell cycle and interrupt the mitosis process [68]. Thus, the cell apoptosis pathway could be activated. Through this approach, these tubulin targeting agents demonstrated their ability to kill tumor cells. Among the known microtubule-binding sites, the colchicine site, localized at the interface of α- and β-tubulin subunits [69], is of particular interest with the discovery of a plethora of colchicine-binding site inhibitors (CBSIs) [70]. The CSBIs scaffolds were guided by the colchicine moiety, which was co-crystallized with α/β-tubulin heterodimers [69]. One of the principal elements of these molecules is a trimethoxyphenyl group, which forms H bonds with Cysteine-241 of the β-tubulin binding site [71]. The other main attribute of CSBIs is a tropolone-derived nucleus that interacts with Threonine-179 and Valine-181 of α-tubulin [72] (Figure 3). Moreover, their pharmacochemical properties (complementary angiogenesis inhibition, good pharmacokinetic parameters, and overcoming multidrug resistance) favor their important rise in the development of novel anticancer compounds [73,74,75].

In view of the effective properties of HDACis and tubulin inhibitors, combination therapy was envisioned with these compounds. In fact, synergistic effects were observed by associating HDACis with tubulin inhibitors (e.g., paclitaxel, vincristine) [76,77]. Therefore, the construction of molecular hybrid HDAC/tubulin inhibitors is the next logical step in the optimization of new-generation anticancer drugs. With all the benefits of the multi-targeting compounds, numerous research groups have studied the HDAC/tubulin dual inhibitors. These dual-targeted molecules would heighten the synergistic antitumor effect observed in the combination therapy, leading to the suppression of tumor cell proliferation and the induction of the apoptosis process in cancer cells.

Given the wide chemical diversity of these biologically active compounds, this review will mainly classify the main dual molecules developed over the last few decades according to the structural scaffolds of the tubulin inhibitors.

## 2. Combretastatin-A4 Motif and Related Structures

**Figure 4:**
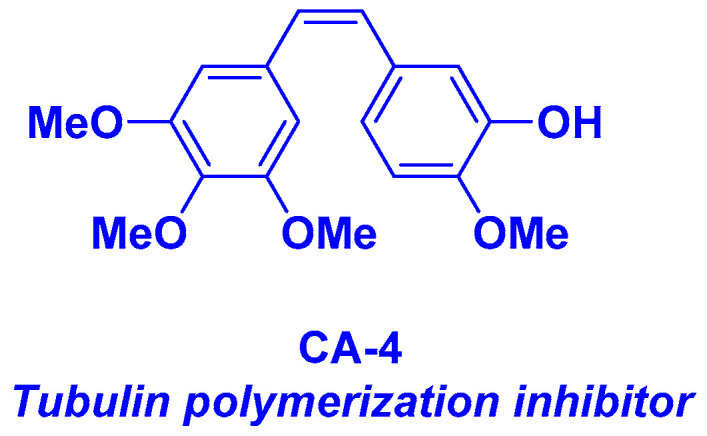
Structure of CA-4.

Combretastatin-A4 (CA-4) is a natural product isolated from *Combretum caffrum*, an African willow tree, by Pettit et al. [78] (Figure 4). This molecule is identified as a potent anticancer agent, which inhibits tubulin polymerization by interacting with the colchicine site (IC_50_ value of 2.62 µM for in vitro tubulin polymerization inhibition) [79]. In addition to its selective action on tubulin, CA-4 possesses a nanomolar-scale cytotoxicity against a broad range of human cancer cells, including multidrug-resistant cells [80]. Despite such promising properties, CA-4 shows high instability of the *Z*-stilbene structure, which could be isomerized during the metabolization process to form the less active *E* isomer. Thus, the development of CA-4 derivatives and related structural analogs was envisioned over the last decades in various medicinal chemistry research groups [81]. By combining these compounds with HDACis, significant synergistic effects were found for these newly designed antitumor agents.

### 2.1. Chalcone Derivatives

**Scheme 1:**
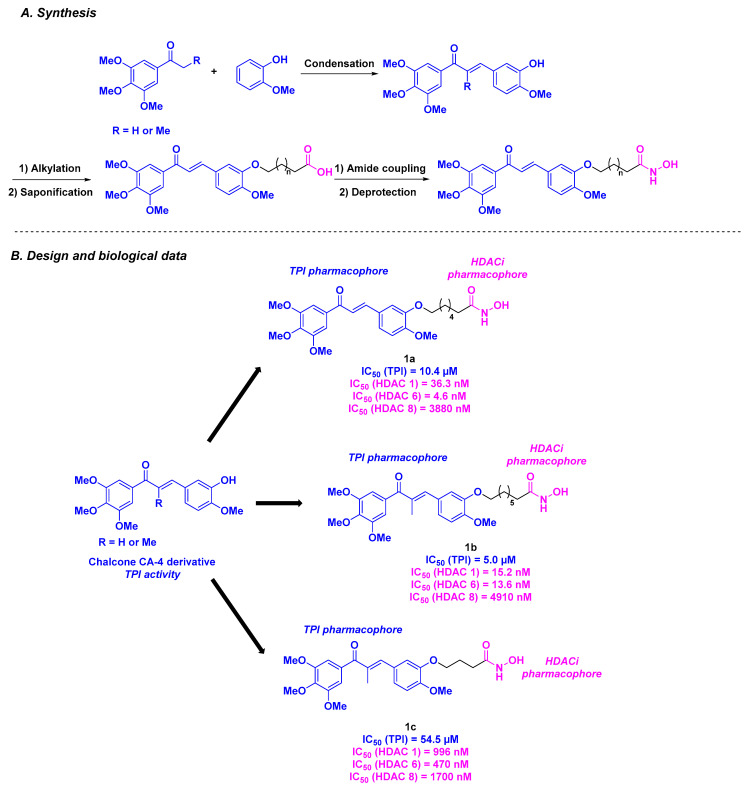
HDAC/tubulin inhibitors designed by Luan et al. [82].

Among the CA-4 related compounds, chalcones constitute one of the main groups. In this manner, Luan et al. described chalcone derivatives as HDAC/tubulin dual-targeting inhibitors. Inspired by the FDA-approved HDACi (see Figure 1) and CA-4 structure, the researchers designed a chalcone platform functionalized with the key methoxy substituents of CA-4, ensuring tubulin inhibition. This part was connected to a hydroxamic acid skeleton, providing HDAC inhibitory activity (Scheme 1). These molecules were constructed through a multi-step synthesis, including a condensation reaction and alkylation step. The HDAC inhibition rates of the prepared hybrid compounds revealed that compounds **1a** and **1b** exhibited the highest inhibitory activities (87% and 92% inhibition at a concentration of 1 µM, respectively). This result was confirmed in HeLa cell nuclear extracts, with IC_50_ values ranging from 71.5 to 132.6 nM for the HDACs. A thorough study showed that **1a** and **1b** have pan-HDAC inhibitory properties, especially with HDAC 1, 6, and 8. After confirming the viability of these molecules as HDACis, their cytotoxicity was evaluated in three cancer cell lines (A549, HeLa, and SGC-7901). Despite its modest HDAC inhibitory activity, molecule **1c** was the most active compound with IC_50_ values of up to 550 nM for A549 cells. Compounds **1a**, **1b**, and **1c** were then subjected to tubulin polymerization inhibition (TPI) assay, with the best inhibition value observed for **1b** (IC_50_ = 5.0 µM). Further exploration of the antiproliferative properties of **1a**, **1b**, and **1c** confirmed their activities against eight human tumor cell lines (LoVo, HCT-116, NCI-H460, NCI-H226, MCF-7, MDA-MB-231, MGC80-3, and BGC-823), especially for **1c** (IC_50_ = 2.40–7.54 µM). More precisely, by analyzing the A549 cells apoptosis, **1c** was able to stop the cell cycle at the G2/M phase at a 10 µM dose. Finally, **1c** also exhibited an anti-migratory effect on A549 cells at a concentration of 5 or 10 µM, as determined by a wound healing assay [82].

**Scheme 2:**
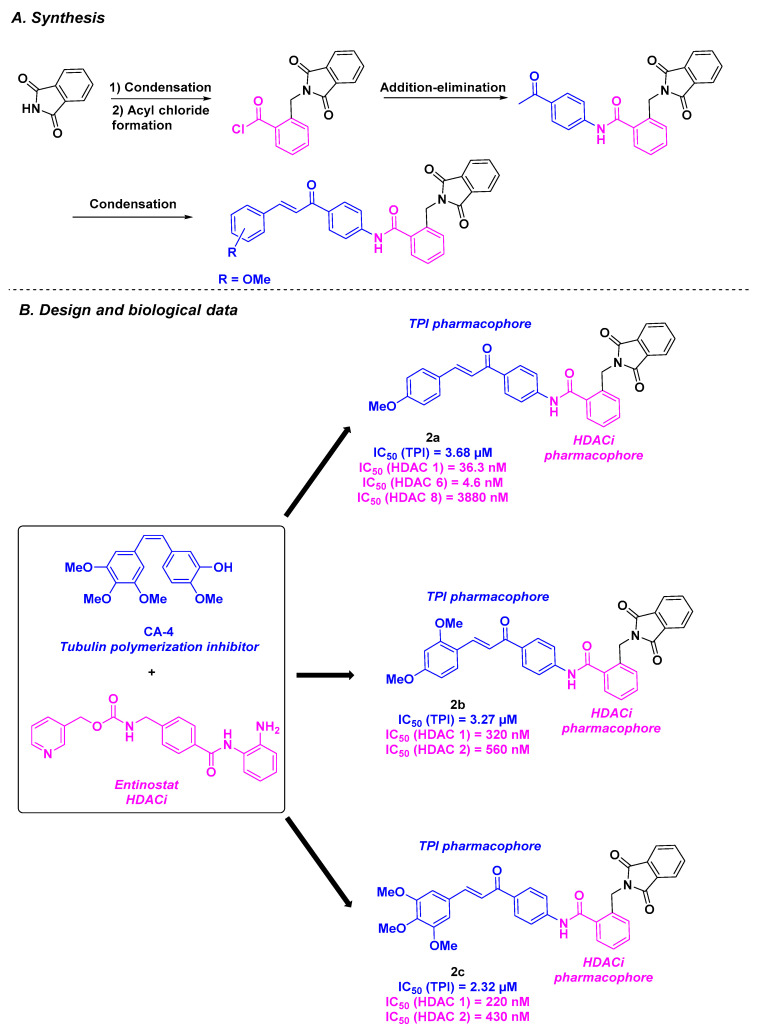
HDAC/tubulin inhibitors conceived by Mourad and his colleagues [83].

Mourad and his colleagues employed analogous scaffolds for their HDAC/tubulin inhibitor hybrids [83]. The group conceived their dual compounds by mixing a chalcone skeleton, which has the same tubulin inhibitory properties as CA-4 [84], with a phthalimide group presenting interesting biological antitumor activities [85]. To ensure HDACi efficiency, a benzamide group was used as an Entinostat analog (Scheme 2). Their chemical synthetic route consists of the condensation of phthalide with potassium phthalimide, followed by addition-elimination and aldolization reactions. In vitro cytotoxicity screening of the prepared molecules revealed that **2a**, **2b**, and **2c** exhibited higher efficacy than CA-4, with IC_50_ values ranging from 1.62 to 2.21 µM against MCF-7 and Hep G2 cells. The TPI capacities of **2a**, **2b**, and **2c** shed light on compounds **2b** and **2c**, which offered the best IC_50_ values of 3.27 and 2.32 µM, respectively. It is worth mentioning that these molecules were selected as hits as their TPI values are comparable to that of CA-4 (IC_50_ = 2.62 µM). Notably, this outcome was in line with the HDAC inhibitory activity of **2b** and **2c**, which had a significant specificity for HDAC 1 and 2. Given the probable involvement of **2b** and **2c** in the cell cycle due to their interaction with tubulin, in vitro DNA flow cytometry was performed. Compounds **2b** and **2c** appeared to trigger apoptosis by stopping cell growth at the G2/M phase. Calculations of energy binding scores confirmed the strong interaction between **2b** and **2c** in the colchicine-binding site of tubulin, mainly due to the hydrogen bonds. Molecular docking investigations of the HDAC active site gave the same conclusions as those noted for the tubulin protein.

### 2.2. Stilbene Derivatives

**Scheme 3:**
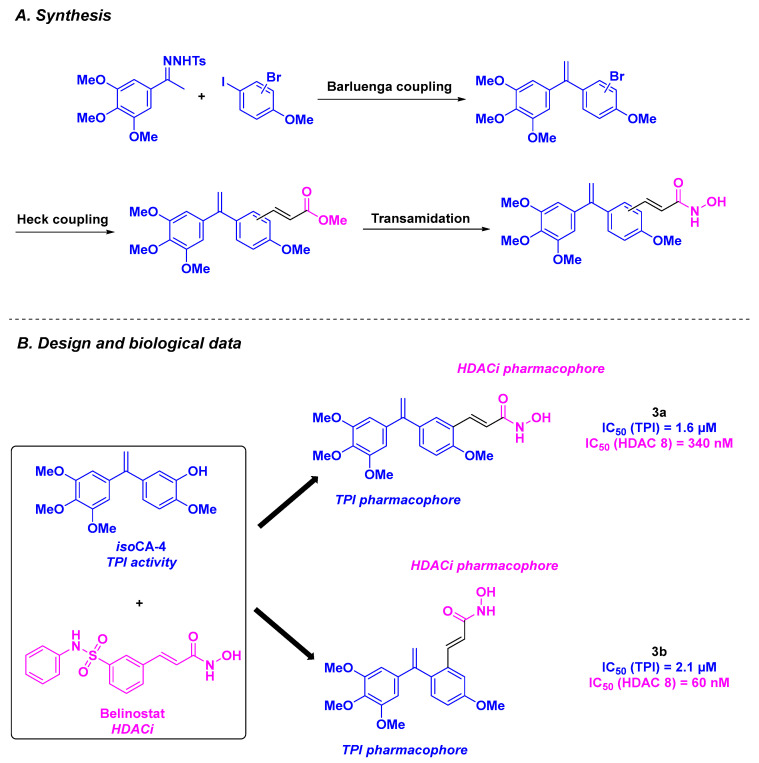
HDAC/tubulin dual inhibitors elaborated by the Hamze group [87].

Inspired by the *iso*CA-4 structure, a stilbene isomer of CA-4 proposed by the Alami group [86], the Hamze team conceptualized novel dual-targeting inhibitors of tubulin and HDAC by associating the *iso*CA-4 moiety with the pharmacophore of belinostat as chimeric HDAC/tubulin inhibitors (Scheme 3). Their chemical synthesis involved a palladium-catalyzed Barluenga-Valdés cross-coupling between an aryl halide and the corresponding *N*-tosylhydrazone. Functionalization with the HDACi pharmacophore (hydroxamic acid) was then realized using different strategies, including alkylation under standard conditions and Sonogashira or Heck couplings. After conducting in vitro antiproliferative assays on HCT-116 cancer cells, the authors validated the importance of the hydroxamic acid group in cytotoxic activity. Compounds with an alkene linker between the stilbene scaffold and hydroxamic acid skeleton exhibited the best GI_50_ values (1.5 nM for **3a** and 8 nM for **3b**). The TPI characteristics of the dual compounds were next determined, with promising IC_50_ values of 1.6 and 2.1 µM for **3a** and **3b**, respectively. The HDAC inhibitory activity of **3a** and **3b** was then determined, with a strong selectivity for HDAC 8. The cytotoxicity of these molecules was also verified in nine cancer cell lines (A549, K562, K562R, PC3, U87-MG, MCF7, BXPC3, MiaPaca2, and HT29), with low GI_50_ values obtained for **3a**, ranging from 0.4 to 5.1 nM. Additional studies by incubating **3a** at a concentration of 5 or 10 nM with HCT-116, K562, and BL2 cells demonstrated the ability of **3a** to stop the cell cycle at the G2/M phase and induce cell death. Finally, the viability of **3a** was corroborated by its safety profile on quiescent peripheral blood lymphocytes, with an IC_50_ value of 7 µM [87].

**Scheme 4:**
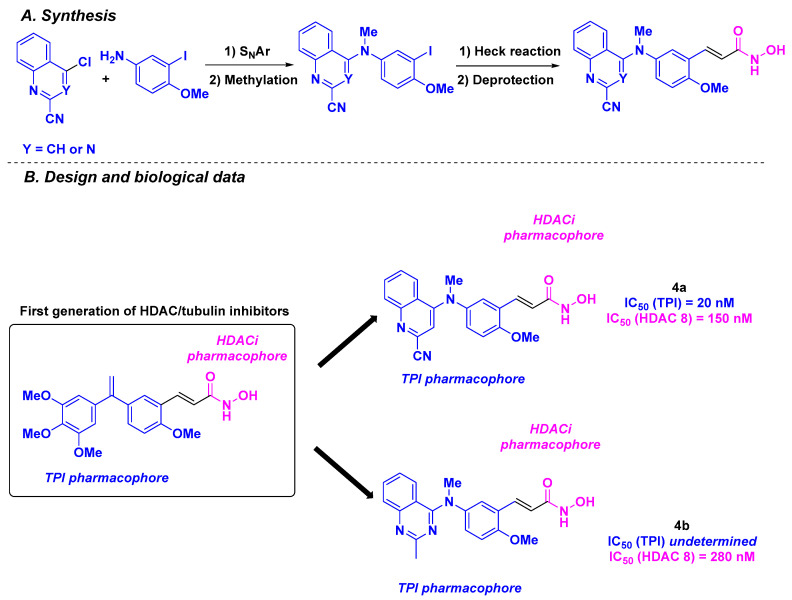
Second generation of HDAC/tubulin dual inhibitors elaborated by the Hamze group [88].

In 2022, the same team proposed a second generation of HDAC/tubulin chimeric inhibitors, by replacing the trimethoxybenzene ring of CA-4 with quinoline or quinazoline cores [88] (Scheme 4). The designed structures were prepared through Buchwald-Hartwig coupling or nucleophilic substitution (S_N_Ar) reaction under acidic conditions, followed by connection to the hydroxamic acid motif via alkylation under basic conditions or through Sonogashira or Heck cross-couplings. The GI_50_ values in HCT-116 cells for the thirty-one synthesized molecules were determined. Structure activity relationship (SAR) studies confirmed the importance of the hydroxamic acid and the alkene linker for cytotoxicity. Thus, **4a** and **4b** were recognized as lead compounds, with GI_50_ values of 0.5 and 0.6 nM, respectively. Encouraged by these outcomes, **4a** and **4b** were tested against nine additional human cancer cell lines (NCI-N87, K562, K562R, MiaPaca2, SKOV3, A549, MCF7, MDA MB231, and HT-29). Notably, **4a** and **4b** remained efficient, with average IC_50_ values of 0.6 and 0.7 nM, respectively. The antiproliferative activities of these second generation of HDAC/tubulin chimeric inhibitors were up to more than 60 times greater than those of the previously described dual molecules, CA-4 and *iso*CA-4. The HDAC inhibitory profiles of **4a** and **4b** were next assessed, showing complete inhibition of HDAC 8 and partial effects on HDAC 6 and 11 (60 to 90% inhibition) at 1 µM. The in vitro TPI assay was performed for **4a**, with an IC_50_ value of 20 nM. Given the crucial role of the microtubule network in cell division, additional flow cytometry analyses revealed cell cycle arrest at the G2/M phase at a concentration of 1 nM of **4a** and **4b**. The authors also demonstrated the involvement of **4a** and **4b** in mitochondrial dysfunction at 2 nM. The metabolic stabilities of **4a** and **4b** were also explored in rat and human microsomes. Compound **4a** exhibited a longer in vitro half-life, likely due to the presence of its quinoline skeleton and hydroxamic acid scaffold. Moreover, the physicochemical properties of **4a** and **4b** were evaluated, with a high water solubility of 68.7 and 65 µg/mL, respectively.

The applicability of **4a** was further validated using an in vivo allograft mouse model. A significant tumor decrease was observed at 0.25 mg/kg and 0.50 mg/kg doses of **4a** injected intratumorally three times per week for over two weeks, without any recidivism after 91 days of treatment. It should be noted that **4a** was solubilized in a PEG vehicle for these assays. No toxicity was observed after the intratumoral injection of **4a** at the above-mentioned doses.

**Scheme 5:**
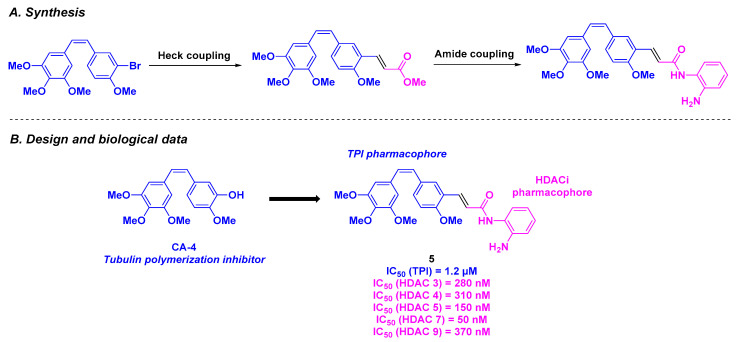
HDAC/tubulin dual inhibitor conceived by Yao, Yang, Duan and coworkers [89].

In 2021, Yao, Yang, Duan and coworkers studied new tubulin/HDAC dual-targeting inhibitors with benzophenones-related and stilbene-derived structures exhibiting antitumor properties [89] (Scheme 5). These compounds were designed through the fusion of CA-4 derivatives (benzophenone or stilbene groups) with HDACi pharmacophores (hydroxamic acid or benzamide moieties). The construction of these dual molecules was based on an alkylation step or palladium-catalyzed Heck cross-coupling, followed by amide bond formation. The generated products were subjected to cytotoxicity assays using six cancer cell lines (MGC-803, MCF-7, U937, A549, HepG2, and HeLa). SAR analysis revealed that stilbene-derived compounds exhibited higher antiproliferative activity than benzophenone analogs. The presence of an alkene linker and benzamide functional group also favored cytotoxicity. Compound **5** was selected as the lead compound, with an IC_50_ ranging from 16 to 305 nM. The specific activity of **5** toward cancer cell lines was confirmed by testing on non-tumorigenic cells (293T, THP-1-derived macrophages, and L02 cell lines), with no observed toxicity (IC_50_ values up to 556 nM). The biological analyses of **5** were pursued with the evaluation of its tubulin polymerization and HDAC inhibitory capacity. Compound **5** was found to be a potent tubulin/HDAC inhibitor, with an IC_50_ value of 1.2 µM for TPI activity, and a pan-HDAC selectivity, especially for HDAC 3, 4, 5, 7, and 9. The TPI phenomenon was also visualized using a confocal microscope by disrupting the cell microtubule network. To explain the high affinity of **5** for its expected targets, molecular docking was performed to detect the binding between **5** and the colchicine site of the tubulin via hydrogen bonds (cf. Figure 3). Similar interactions were observed between **5** and HDAC 7. By deepening the understanding of the mechanism of action of **5**, the research group discovered that **5** induced cell cycle arrest at the G2/M phase by upregulating Cyclin B1 and diminishing p21 and p-CDC2 expression. Compound **5** also contributed to cell death by decreasing the concentration Caspase-3, Caspase-7, Caspase-9 and PARP (Poly(ADP-Ribose) Polymerase) and increasing the levels of cleaved corresponding proteins. In addition to this pro-apoptotic pathway, compound **5** altered mitochondria membrane potential and promoted the generation of reactive oxygen species (ROS). A wound healing assay using human umbilical vein endothelial cells (HUVECs) revealed that **5** inhibited cell migration, invasion, and angiogenesis.

Finally, the effectiveness of **5** was tested in zebrafish embryos, where angiogenesis was impeded at 0.6 µM of **5**. Using xenograft zebrafish models, a significant tumor reduction was observed after incubation with **5** at a concentration of 100 nM.

**Scheme 6:**
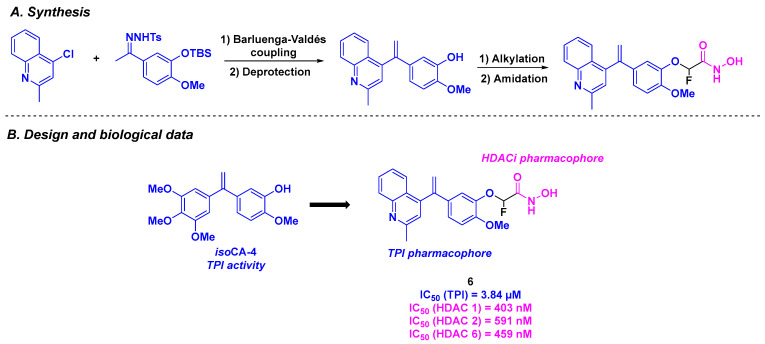
HDAC/tubulin dual inhibitor proposed by Chen, Xu and their colleagues [90].

Similarly, the CA-4 derivatives developed by Chen, Xu, and their colleagues featured a stilbene skeleton bearing a quinoline or pyridine motif to enhance tubulin polymerization inhibitory activity. To fulfill the dual properties of these molecules, the research team incorporated a hydroxamic acid structure into the stilbene scaffold as an HDACi [90] (Scheme 6). The construction of the stilbene moiety relied on a Barluenga-Valdés cross-coupling. The hydroxamic acid group was installed via alkylation or a Wittig reaction, followed by amide bond formation. By evaluating the antiproliferative activities of the synthesized dual compounds, compound **6** exhibited the best in vitro IC_50_ value against K562 cells (3 nM). Further investigations confirmed the superior cytotoxicity of **6** in five cancer cell lines (MCF-7, MDA-MB-231, A549, B16F10, and A2780), with IC_50_ values ranging from 5 to 36 nM. In contrast, **6** demonstrated low cytotoxicity in normal human lung cells (IC_50_ (HFL-1) = 230 nM). The HDAC inhibitory character of **6** was validated, with pan-HDAC selectivity, especially for HDAC 1, 2, and 6. The TPI effect of compound **6** was also established in vitro, with a measured IC_50_ value of 3.84 µM. Competitive inhibition experiments with tritium-labeled colchicine indicated that **6** acts by binding to the colchicine site of tubulin. The dual characteristic of **6** was confirmed in K562 cells, which showed disruption of the microtubule network and accumulation of acetylated α-tubulin. In cellulo assays showed that **6** was able to stop the cell cycle at G2 phase and induce the apoptosis of K562 cells by increasing the concentration of pro-apoptotic proteins Bad and Bax. Compound 6 also exhibited antivascular activity, suppressing HUVECs migration at 8 nM. Finally, an in vivo antitumor assay was performed with **6**, using a mouse liver cancer allograft model. Similar to the in vitro results, **6** exhibited excellent efficacy (tumor growth inhibition (TGI) = 73.12% at 20 mg/kg daily for 17 days, by intravenous injection in vehicles containing DMSO (10%), Tween 80 (2%), and saline (88%)), without loss in body weight or significant toxicity in vital organs.

### 2.3. Amine Derivatives

**Scheme 7:**
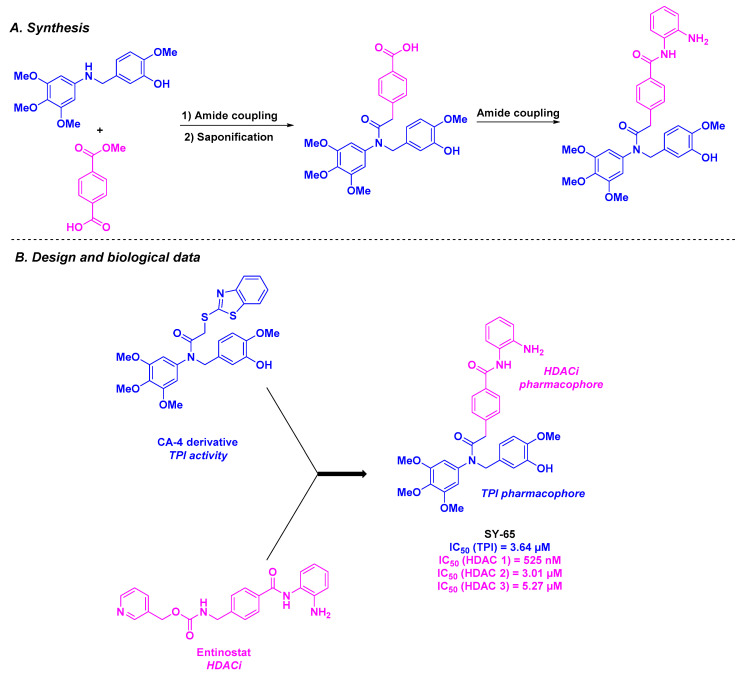
HDAC/tubulin inhibitor **SY-65** prepared by Song, Zhang and coworkers [91].

In 2022, Song, Zhang, and coworkers combined the CA-4-derived skeleton with the key motif of Entinostat, an HDACi, to construct **SY-65**, a novel aromatic amide scaffold targeting HDAC1 and tubulin (Scheme 7). The synthesis of **SY-65** involved a three-step process consisting of two amide couplings and a saponification reaction. This compound exhibited activity against several human cancer cell lines (MGC-803, HGC-27, SGC-7901, HCT-116, and KYSE450), with IC_50_ values ranging from 39 to 254 nM. With these promising results, **SY-65** was then studied as a tubulin polymerization inhibitor in MGC-803 and HGC-27 cells, showing an IC_50_ of 3.64 µM. Complementary *N*,*N*′-ethylene-bis(iodoacetamide) (EBI) competition and cellular thermal shift assays confirmed that **SY-65** binds to the colchicine site on β-tubulin. By evaluating the activities of **SY-65** against HDACs, the authors reported the inhibition of HDAC1 (IC_50_ = 525 nM). This effect was also observed in cellulo with MGC-803 and HGC-27 cell lines. In addition, **SY-65** exhibited apoptotic properties in MGC-803 and HGC-27 cells, including the reduction of Bcl-xL, Bcl-2, and c-IAP1 levels. This compound also arrested cell proliferation at the G2/M phase in these cell lines, as evidenced by the downregulation of the cell cycle-related proteins Wee1, cdc2, and phosphorylated cdc2. An in vivo nude mouse xenograft model bearing MGC-803 cells confirmed all the biological features of **SY-65**, especially the inhibition of tumor growth after 14- or 21-day treatment through daily intraperitoneal 30 mg/kg injection [91].

### 2.4. Oxazole Derivatives

**Scheme 8:**
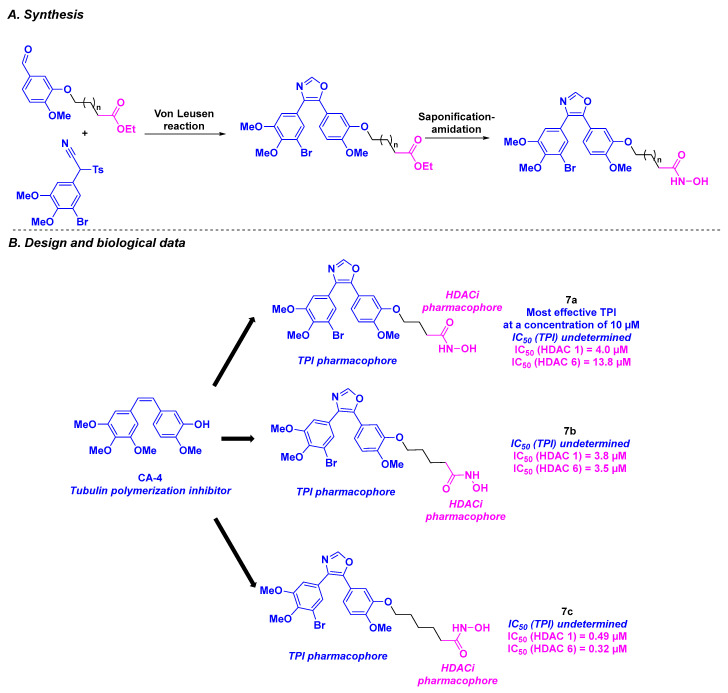
HDAC/tubulin inhibitors prepared by Höpfner et al. [92].

In 2019, Höpfner et al. created novel HDAC/tubulin inhibitors by attaching hydroxamic acids to oxazole-bridged CA-4 derivatives (Scheme 8). These bent structures were prepared via a three-step synthesis, with the Von Leusen reaction under basic conditions as the key step. The biological evaluation of these hybrid compounds revealed that bromo derivatives **7a**, **7b**, and **7c** exhibited the highest average antiproliferative activities for six cancer cell lines (518A2, 518A2, HT-29, DLD-1, HCT-116, KB-V1^Vbl^, MCF-7^Topo^) with IC_50_ values ranging from 0.11 to 14.2 µM. It should be noted that **7a**–**c** were also active against Ea.Hy926 cells (endothelial hybrid cells), indicating their potential antivascular capacity as CA-4. In addition, **7a**–**c** showed strong selectivity toward cancer cells, in contrast to their lack of major cytotoxicity in non-malignant HDFa dermal fibroblasts.

Tubulin polymerization assays were then performed with **7a**–**c**, revealing **7a** as the most effective tubulin polymerization inhibitor at a concentration of 10 µM. Similar results were observed in 518A2 melanoma cells, with disruption of the microtubule network at 0.5 µM of **7a**. The HDAC inhibitory properties of **7a**–**c** were studied next. Although **7a** was the most effective compound against cancer cells and microtubules, only moderate HDAC 1 and HDAC 6 inhibition was observed. In contrast, **7c** exhibited high specificity for HDAC 1 and HDAC 6, with IC_50_ values of 0.49 and 0.32 µM, respectively. These data were validated by molecular docking with stronger affinities for HDAC 1 and HDAC 6 for molecules bearing long alkyl linkers (**7c** vs. **7a**). Western blot analyses highlighted that **7c** could also impede the acetylation of tubulin, without disturbing the microtubule skeleton, due to its concomitant HDAC 6 inhibitory activity. The FACS (Fluorescence-activated cell sorting) data on 518A2 melanoma cells corroborated the effect of **7a**–**c** on the cell cycle: **7a** and **7b** led to the interruption of cell growth in the G2/M phase, while **7c** caused arrest in the G1 phase. The proof of concept was then verified by determining the toxicity of **7a** in an in vivo nude mouse model, with a formulation of **7a** containing 10% Tween 80, 10% ethanol, and 80% saline. The treatment was well tolerated at high doses (up to 200 mg/kg orally administered once for two weeks) without causing weight loss [92].

### 2.5. 1,4-Diarylazetidin-2-one Derivatives

**Scheme 9:**
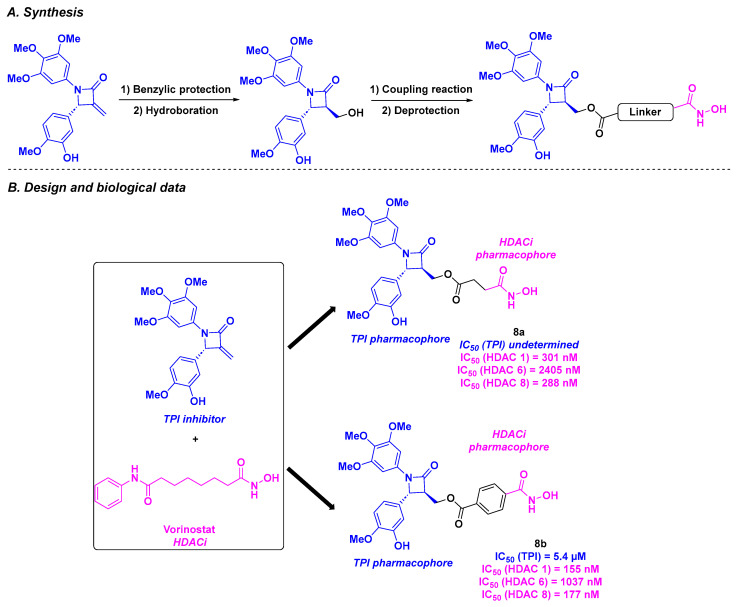
HDAC/tubulin inhibitors designed by Ding, Wang and coworkers [94].

Based on the chiral 1,4-diarylazetidin-2-one structure, a constrained analog of CA-4, capable of inhibiting tubulin by binding the colchicine site [93], and the vorinostat skeleton, Ding, Wang, and coworkers designed an original skeleton as a chimeric inhibitor [94] (Scheme 9). The synthesis of these molecules began with benzylic protection and hydroboration. The alcohol intermediate was subsequently condensed with the corresponding carboxylic acid to afford the desired product. In vitro cytotoxicity studies against four tumor cell lines (BE-(2)-C, A549, U87MG, and HCT-116) revealed that compounds **8a** and **8b** exhibited the best antiproliferative activities, with IC_50_ values ranging from 16 to 56 nM. Further exploration of the HDAC isoform inhibitory capacity demonstrated a high selectivity toward HDAC 1 and HDAC 8 for compounds **8a** and **8b**. It should be noted that **8b** exhibited better IC_50_ values against HDAC 1 and HDAC 8 compared to **8a**. The HDAC inhibitory property of **8b** was also confirmed in cellulo through western blot analyses, by the detection of non-deacylated substrates of HDAC 1 and HDAC 8. Compound **8b** also acted as a proven TPI, with an IC_50_ value of 5.4 µM for in vitro tubulin polymerization inhibition. Molecular docking supported these results, with a tight fixation of **8b** via hydrophobic interactions between the methoxyphenyl groups and the apolar binding site of tubulin (cf. Figure 3). The interaction between **8b** and HDAC 8 was ascertained by the chelation of the hydroxamic moiety with Zn^2+^ localized in the active pocket and the hydrogen bonds with key amino acids (Tyr341, His141, and His142). Additionally, **8b** could inhibit the cancer cell colony formation at a concentration of 100 nM. A thorough inspection of **8b**’s biological pathway suggested that **8b** induced G2/M mitosis arrest and caused the cell apoptosis by activating the cleavage of pro-apoptotic proteins PARP-1 and Bax. The microsomal stability of **8b** was also evaluated, revealing a long half-life (t_1/2_ = 68.8 min) and an intrinsic clearance of 18.1 mL/min/kg in human liver microsomes. A high water solubility was also found for **8b**, with a value of 102.2 µg/mL. The antitumor activities of **8b** were endorsed in human neuroblastoma xenograft models, with a significant decrease in tumor growth at an intraperitoneal dose of 25 mg/kg every three days, leading to a TGI of 67%. Notably, **8b** demonstrated synergistic efficiency, with better TGI values than those observed, with paclitaxel and vorinostat used alone. The in vivo low toxicity of **8b** was verified in nude mice, with minor weight loss and adverse effects noted.

### 2.6. Arylpyridine Derivatives

**Scheme 10:**
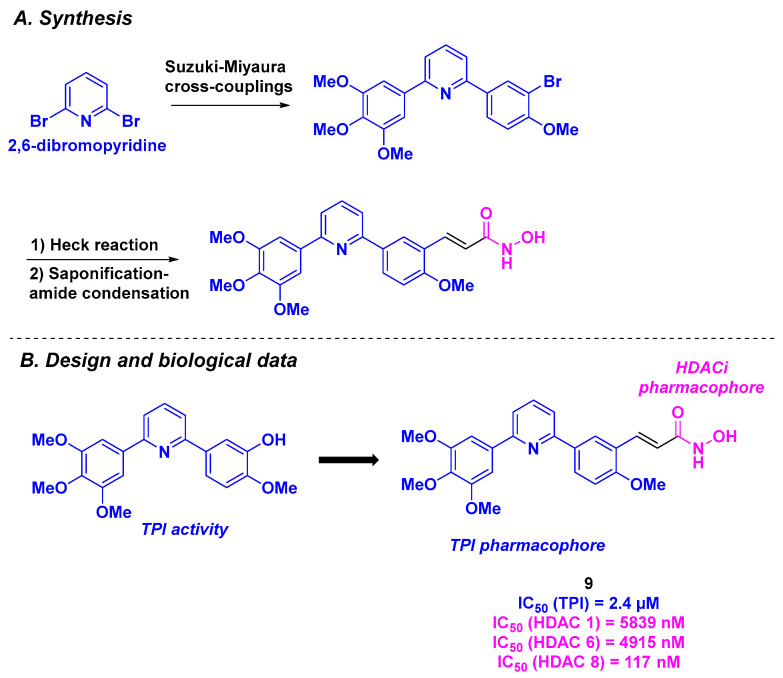
HDAC/tubulin inhibitor designed by Ding, Wang and coworkers [95].

In 2022, Ding, Wang, and their colleagues proposed a new dual antitumor agent presenting a diarylpyridine scaffold (Scheme 10) [95]. The rational design of these structures relied on the association between pyridine-bridged analogs of CA-4, which exhibited comparable biological properties to CA-4 [96], with hydroxamic acid as the HDACi motif. The synthesis of these frameworks involved the use of commercially available 2,6-dibromopyridine, which underwent two Suzuki-Miyaura cross-couplings to form the corresponding diarylpyridine. The branching of the HDACi pharmacophore was established via an alkylation step or a Heck coupling, followed by amide condensation. The resulting molecules were then subjected to diverse in vitro biological assays. After screening for antiproliferative activities against four cancer cell lines (BE-(2)–C, A549, U87MG, and HCT-116), compound **9** was selected as the hit compound, with IC_50_ values ranging from 17 to 90 nM. The dual properties of **9** were then examined. On one hand, **9** presented a high specificity for HDAC 8 (IC_50_ = 117 nM). On the other hand, **9** significantly inhibited tubulin polymerization, with an IC_50_ value of 2.4 µM. In cellulo experiments showed a disruption of the microtubule network at a concentration of 100 nM of **9**. By affecting the microtubule structure, **9** could also arrest cell mitosis at the G2/M phase by upregulating the expression of Cyclin-1 and promoting the hyper-phosphorylation of key mitotic proteins (Bubr-1 and Histone 3). **9** could also participate in the apoptosis process by inducing the formation of cleaved pro-apoptotic markers (PARP-1 and Bax). The evaluation of the metabolic stability in liver microsomes of **9** showed better half-life and clearance values than CA-4 (54.8 min and 22.8 mL/min/kg for **9** vs. 13.6 min and 89.5 mL/min/kg for CA-4). The water solubility of **9** was determined in PBS buffer, with a good value of 138 µg/mL. Finally, compound **9** was tested in vivo using a human neuroblastoma xenograft mouse model. At a unique intraperitoneal dose of 50 mg/kg every three days, **9** had a TGI of 61%, which was higher than those observed for paclitaxel or vorinostat employed alone (N. B.: TGI = 30% at 5 mg/kg of paclitaxel and TGI = 19% at 50 mg/kg of vorinostat). In comparison with the combination of paclitaxel/vorinostat, the TGI of **9** remained higher (61% vs. 44%), demonstrating the synergistic effect of dual compound **9**.

**Scheme 11:**
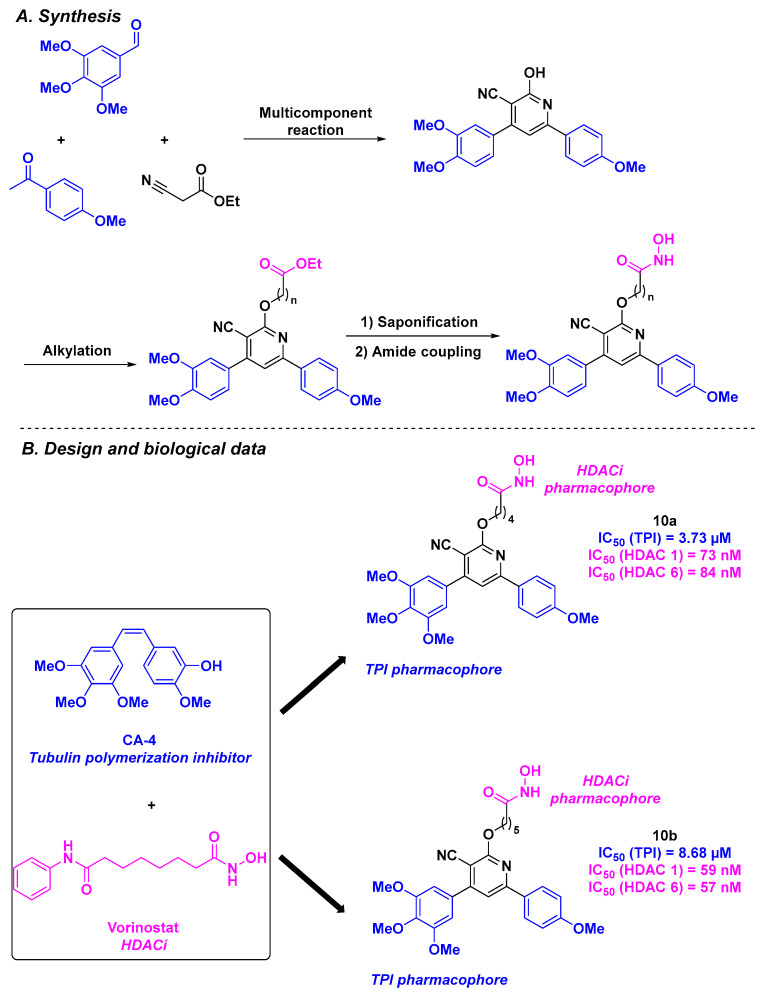
HDAC/tubulin inhibitors synthesized by Abdelhafez et al. [97].

More recently, Abdelhafez et al. constructed a library of dual inhibitors by combining the key motifs of CA-4 with the pharmacophore of vorinostat. The linkage between the main functional groups was ensured by a central cyanopyridine core [97] (Scheme 11). The chemical approach for the preparation of these scaffolds consisted of a multicomponent reaction between 4-methoxyacetophenone, 3,4,5-trimethoxybenzaldehyde, and ethyl cyanoacetate. The cyanopyridine derivatives were then subjected to an alkylation reaction and an amide coupling. The antiproliferative assays allowed the identification of the hit compound **10a**, which exhibited the best IC_50_ values for UO-31 (IC_50_ = 3.52 µM) and T47D cell lines (IC_50_ = 5.50 µM). The HDAC inhibitory activity of the hybrid compounds was then quantified, highlighting that **10b** was more efficient than **10a**, especially for HDAC 1 and 6. The TPI assay emphasized that **10a** has an IC50 value comparable to that of vinblastine, an anticancer agent with potent TPI capacity (IC_50_ values of 3.73 and 2.86 µM, respectively). The cytotoxicity of **10a** and **10b** on WI-38 normal cells was also assessed, showing only minor effects (IC_50_~30 µM). After obtaining satisfactory results with **10a**, its mechanism of action was elucidated through complementary biological studies. Thus, the authors pointed out that **10a** favored the cell cycle arrest at the G2/M phase, leading to the apoptosis process. Western blotting showed that the cells were arrested in the G2/M phase by **10a**. This outcome demonstrated the clear influence of **10a** on cell mitosis arrest via a biological pathway involving HDAC and tubulin.

## 3. Colchicine Derivatives

Colchicine, a natural product extracted from the corms of *Colchicum autumnale* L., is one of the first potent tubulin destabilizers known. Alongside its therapeutic use in familial Mediterranean fever and gout, this compound is also an efficient anticancer agent that inhibits mitosis in diseased cells. Although colchicine efficacy has been well demonstrated in tumor cell lines, its therapeutic index remains narrow. This major drawback results in significant side effects, such as anemia, neutropenia, and bone marrow damage [68].

To overcome these issues, intensive research on colchicine-related molecules has been conducted over the last decade. In this context, tubulin/HDAC dual-targeting inhibitors have been developed.

**Scheme 12:**
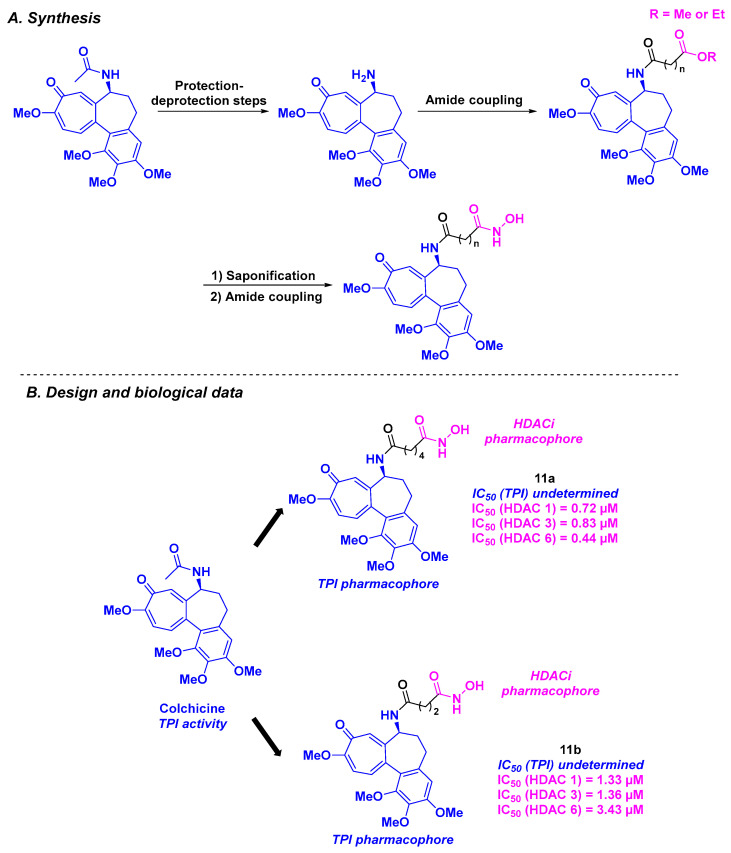
HDAC/tubulin inhibitors designed by Lu, Chen, and coworkers [98].

In 2013, Lu, Chen, and coworkers reported the design and synthesis of tubulin/HDAC inhibitors based on colchicine and hydroxamic acid scaffolds (Scheme 12). The synthetic route for these dual molecules relied on a three-step synthesis, including two amidation reactions mediated by HATU (Hexafluorophosphate Azabenzotriazole Tetramethyl Uronium). The HDAC inhibitory properties of the five elaborated compounds were studied, showing pan-HDAC selectivity for HDAC 1, 3, and 6, especially for compound **11a**, with IC_50_ values ranging from 0.44 to 0.83 µM. By treating BEL-7402 cells with all these tubulin/HDAC inhibitors, the authors noticed that **11b** significantly promoted cell cycle arrest at the G2/M phase at a concentration of 1 µM. The viability of the hybrid molecules was validated by exploring their antiproliferative activities against five cancer cell lines (A431, A549, HCT-116, MCF-7, and PC-3). Compound **11b** was thus the most active compound, with an IC_50_ ranging from 0.242 to 4.672 µM [98].

**Scheme 13:**
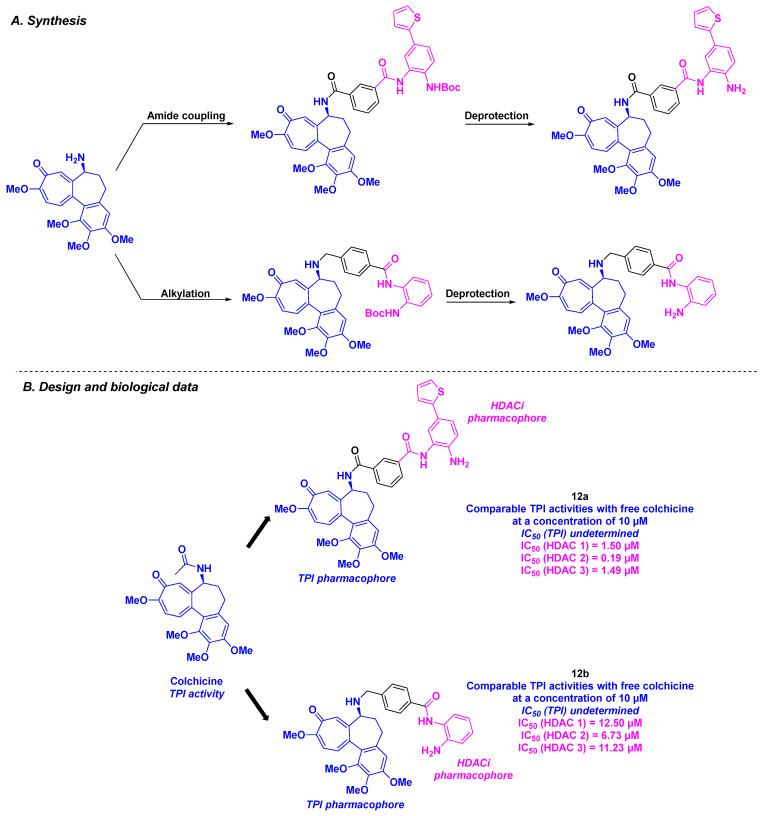
HDAC/tubulin inhibitors described by Fang, Lu and coworkers [99].

Among the colchicine derivatives employed as HDAC/tubulin inhibitors, the hybrid compounds developed by Fang, Lu, and coworkers merged a colchicine core with TPI activity and a benzamide motif for the HDACi property (Scheme 13). The construction of these hybrid structures was based on amide coupling using HATU as the coupling agent or an alkylation step between the two main chemical entities. The in vitro HDAC inhibitory activity was evaluated for all the synthesized compounds, emphasizing that compound **12a** was the most active molecule against HDAC 1, 2, and 3, with IC_50_ ranging from 0.19 to 1.50 µM. The TPI effect was confirmed for all the prepared hybrid inhibitors, namely **12a** and **12b**, which possessed activities comparable to free colchicine at a concentration of 10 µM. These outcomes corroborated with the cell cycle analyses, where **12a** and **12b** were found to block mitosis at the G2/M phase of the cell cycle. The cytotoxicities of **12a** and **12b** were also explored. Compound **12b** exhibited higher antiproliferative activity towards twelve human cancer cell lines (A549, HCT-116, SW620, Hep3B, HeoG2, MHCC97H, SNU-5, SNU-16, MKN-45, PANC-1, and SJSA-1) compared to **12a**, with IC_50_ values ranging from 2 to 106 nM [99].

## 4. Aminobenzamide Core

**Scheme 14:**
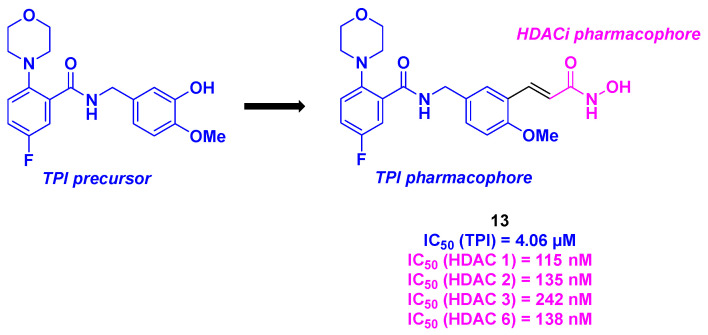
HDAC/tubulin hybrid inhibitor bearing an aminobenzamide core developed by the Xu group [101].

Based on their previous outcomes with tubulin inhibitors [100], Xu group reported in 2022 the elaboration of dual molecules displaying an aminobenzamide core capable of inhibiting microtubule polymerization combined with a hydroxamic acid core as an HDACi (Scheme 14). In vitro antiproliferative assays showed that compound **13** exhibited significant IC_50_ values (26–30 nM) in six cancer cell lines (HepG2, HCT-116, MDA-MB-231, H22, and MCF-7). Satisfactory results were obtained for the in vitro inhibitory potencies against HDACs of compound **13** against HDACs, with an inhibition rate of 93% at 10 µM. Further investigations around the HDAC subtype validated the pan-HDAC-inhibiting property of molecule **13**, with a selectivity toward HDAC 1, 2, 3, and 6. Moreover, compound **13** exhibited TPI activity with an IC_50_ value of 4.06 µM, particularly in the colchicine-binding site. The concomitant role of compound **13** was validated in HepG2 cells, where the HDAC/tubulin inhibitory effects were identified using western blot analysis. Mechanistic insights revealed that molecule **13** could induce cell death through several pathways, such as its ability to stop the cell cycle at the G2/M phase, activate the expression of pro-apoptotic Bad and Bax proteins, induce mitochondrial membrane potential depolarization, or increase ROS levels. Notably, similar to its TPI precursor, compound **13** exhibited significant antivascular activity in HUVECs. Finally, the in vivo antitumor properties of **13** were evaluated using a liver cancer allograft mouse model. A major reduction in tumor weight by 82% (at an intravenous dose of 20 mg/kg per day through a vehicle featuring DMSO (10%), Tween 80 (2%), and saline (88%) for a three-week treatment) was observed in the presence of **13**, corroborating all the in vitro efficacies [101].

## 5. Amide Derivatives

**Scheme 15:**
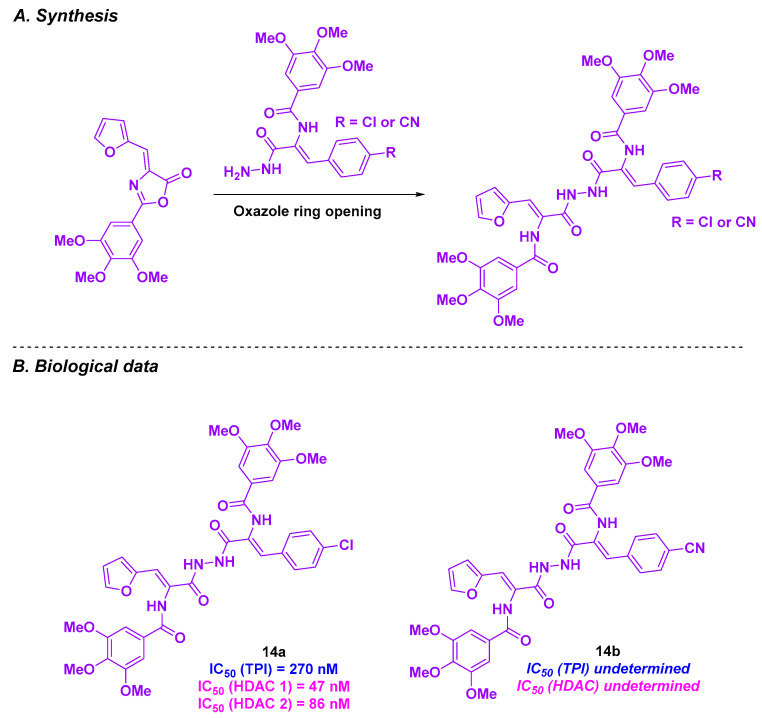
HDAC/tubulin hybrid inhibitors created by Zaki and coworkers [102].

Zaki and coworkers created a novel series of HDAC/tubulin inhibitors by integrating amide functional groups [102] (Scheme 15). The synthesis of these compounds relied on an oxazole ring-opening with the corresponding aniline or hydrazide. The conceived products were then biologically evaluated, and the best IC_50_ values against HepG2 cells were observed for **14a** and **14b** (0.65 and 0.92 µM, respectively). In contrast, low toxicities were reported for **14a** and **14b** normal liver HL-7022 cell lines (IC_50_ values of 9.62 and 11.09 µM). Compound **14a** exhibited higher HDAC inhibitory activity than **14b**, with IC_50_ values ranging from 47 to 86 nM for HDAC 1 and HDAC 2. A TPI assay was then performed for **14a**, yielding an IC_50_ value of 270 nM. This TPI effect was validated in cellulo, with cell division arrest at the G2/M phase. This phenomenon could lead to HepG2 cell apoptosis after incubation with **14a** at a concentration of 0.65 µM for 48 h. This apoptosis pathway was activated through the upregulation of caspases 3 and 7. As caspases 3 and 7 are key markers of apoptosis in mitochondria, the analysis of mitochondrial events was conducted. It was noticed that **14a** was able to activate caspases 3 and 7, resulting in mitochondrial apoptosis.

## 6. Quinolone Derivatives

**Scheme 16:**
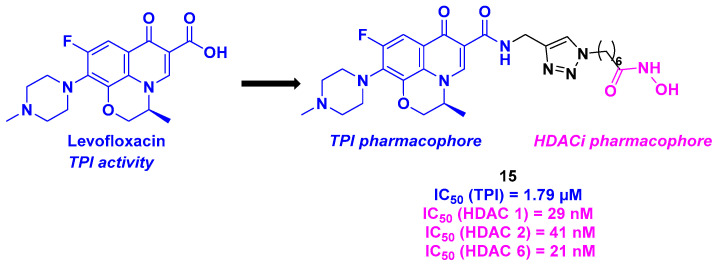
Dual HDAC/tubulin inhibitor conceived by Wang et al. [104].

In 2018, Wang et al. conceived a novel series of dual compounds using a quinolone derivative as TPI attached to a hydroxamic acid side chain as HDACi through a triazole linkage (Scheme 16). More precisely, the group designed a TPI pharmacophore based on the interesting properties of levofloxacin derivatives as antitumor agents [103]. The HDAC inhibition activity was first investigated. The prepared dual conjugates presented high affinity for HDAC 1, 2, and 6, especially molecule **15**, with IC_50_ values ranging from 21 to 41 nM. TPI evaluation of the hybrid conjugates showed that all the synthesized compounds were more effective than levofloxacin. As previously reported, compound **15** exhibited the highest efficacy, with an IC_50_ value of 1.79 µM. After the investigation of the in vitro properties of the dual molecules, the whole cell antiproliferative activity was examined. Again, **15** confirmed its antitumor capacity against five cancer cell lines (A549, HepG2, MCF-7, PC-3, and HeLa), with IC_50_ values ranging from 0.3 to 4.9 µM. Finally, the viability of HDAC/tubulin inhibitors was established in the healthy epithelial cell line MCF-10A, where negligible toxicity was observed for all these compounds [104].

## 7. Benzofuran Scaffold

**Scheme 17:**
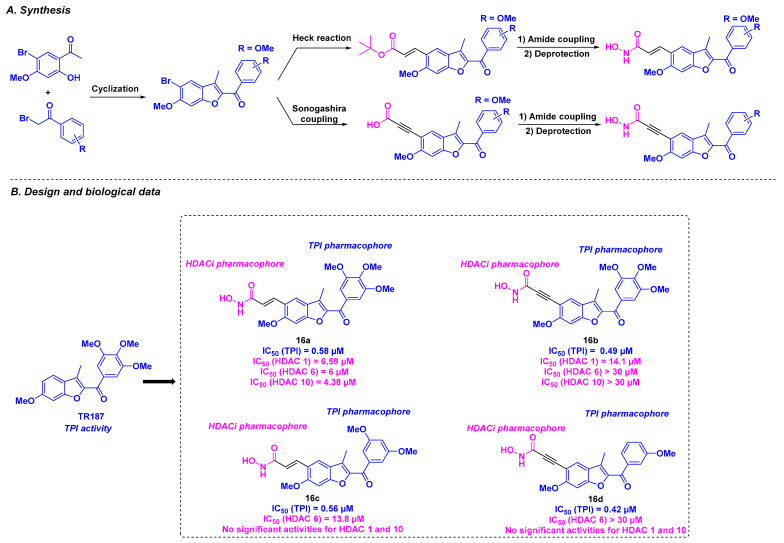
Dual HDAC/tubulin inhibitors developed by the Romagnoli group [106].

Based on their previous work on benzofuran derivatives as inhibitors of tubulin polymerization [105], the Romagnoli group envisaged the development of dual molecules using their lead compound **TR187**, which binds to the tubulin colchicine site and a hydroxamic acid as an HDACi pharmacophore [106] (Scheme 17). These compounds were synthesized from diversely substituted α-bromo acetophenones, which underwent a cyclization reaction under basic conditions to afford the benzofuran core. The connection with the hydroxamic group was carried out via Heck or Sonogashira coupling. The molecular hybrids thus prepared were subjected to different biological tests. The cytotoxicity studies led to the identification of **16a** and **16b** as hit molecules, exhibiting promising IC_50_ values ranging from 0.4 to 23.5 nM against HeLa, MDA-MB-231, A549, HT-29, and MCF-7 cells. Despite these interesting results, the TPI assay demonstrated that **16c** and **16d** had the best tubulin inhibitory potencies, with IC_50_ values of 0.56 and 0.42 μM, respectively. Although **16a** and **16b** were less effective (IC_50_ values of 0.58 and 0.49 µM, respectively), these compounds remained more potent than CA-4. The ability of these molecules to inhibit HDACs revealed only moderate activity toward HDAC 1, 6, 8, and 10. Compound **16a** exhibited pan-HDAC specificity for HDAC 1, 6, and 10, whereas **16b** was more selective for HDAC 1, and **16c** had a strong affinity for HDAC 6. In contrast, negligible HDAC inhibitory capacity was found for **16d**. Based on these outcomes, the authors suggested that the antiproliferative activities of these compounds might mainly result from the TPI effect.

## 8. 2-Benzylideneindanone Scaffold

**Scheme 18:**
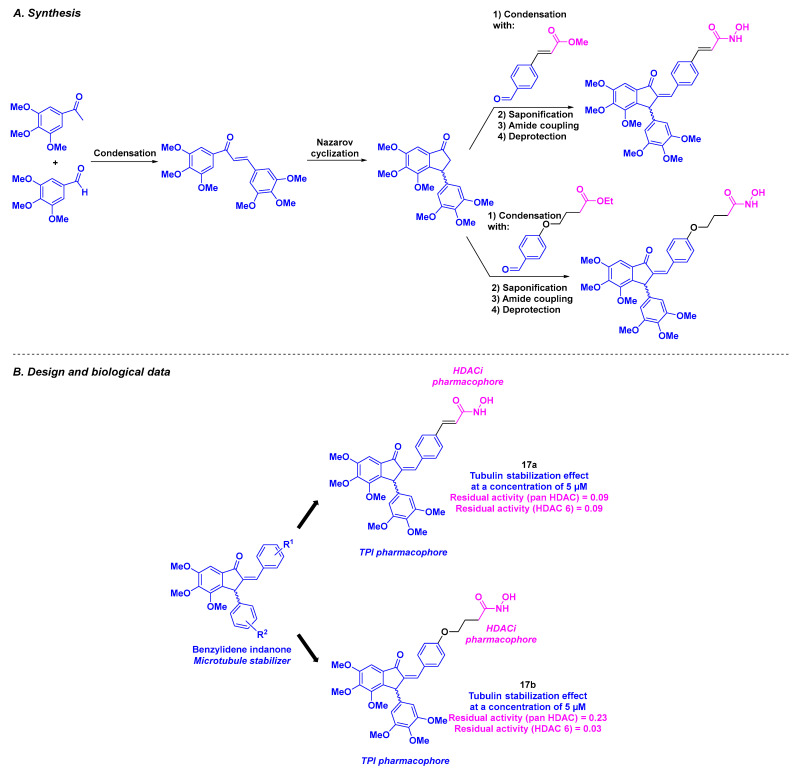
Dual HDAC/tubulin inhibitors described by Negi et al. [110].

With their long-lasting interest in indanone and benzylideneindanone derivatives as potential anticancer agents [107,108,109], Negi et al. reported a hybrid inhibitor bearing a hydroxamic acid skeleton as an HDACi joined to an indanone core for TPI activity (Scheme 18). The installation of the benzylideneindanone moiety involved a condensation reaction under basic conditions between 3,4,5-trimethoxybenzaldehyde and trimethoxyacetophenone, followed by Nazarov cyclization. The linkage with the hydroxamic acid core was provided by condensation, saponification, amidation, and a final deprotection step. The synthesized benzylideneindanone derivatives were subjected to antiproliferative studies against three human cancer cell lines (MCF-7, MD-AMB-231, and K562). Compounds **17a** and **17b** were selected for further biological assays based on their satisfactory IC_50_ values (IC_50_ = 0.36–49.67 µM). Their low cytotoxicity toward normal Vero cells (IC_50_ = 100.32 µM and IC_50_ = 47.23 µM for **17a** and **17b**, respectively) validated their selectivity for malignant cell lines. Cell cycle analyses in the presence of **17b** showed that **17b** could promote apoptosis by blocking the G2/M and S phases. Further mechanistic insights indicated a major stabilization effect of tubulin by **17a** and **17b** at 5 µM. This phenomenon was also observed in confocal microscopy as a disorganization of the cytoskeletal tubulin network. Finally, the authors described the pan-HDAC inhibitory activity of **17a** and **17b**, with a high specificity of **17b** for HDAC 6 at 20 µM, attesting to the dual character of these compounds. The aqueous solubility of **17b** was also measured, affording a moderate value of 1.76 µg/mL.

In addition, **17a** and **17b** exhibited potential anti-inflammatory properties in macrophage cells, with a significant reduction at 10 µM in the rates of TNF-α and IL-6 (12 to 29%), which are indicators of cancer inflammation. Complementary safety aspects were explored for **17a** in mice at various concentrations (5–1000 mg/kg via a single oral administration), identifying **17a** as a tolerable and relatively safe agent [110].

## 9. Aminothiazoles

**Scheme 19:**
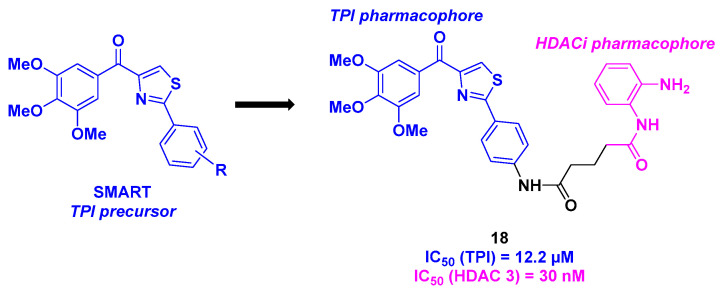
Dual HDAC/tubulin inhibitor prepared by Chen et al. [112].

Building on their recent discoveries on methoxybenzoyl-aryl-thiazole (SMART) derivatives as TPI agents [111], Chen et al. prepared a new class of dual HDAC/tubulin inhibitors in 2021 (Scheme 19). By associating the SMART core with a benzamide scaffold as the HDACi pharmacophore through a multi-step synthesis, a wide range of dual compounds were elaborated and employed in various biological assays. First, the cytotoxic activities of these molecules were evaluated, leading to the identification of compound **18**, which was highly potent against HCT-116, B16-F10, Jurkat, and A549 cancer cells (IC_50_ = 30–140 nM). **18** validated its on-mechanism cytotoxicity in an HDAC inhibitor-resistant cancer cell line (YCC3/7) with an IC_50_ value of 560 nM. The analysis of the inhibition of HDAC isoforms demonstrated the selectivity of **18** toward HDAC 3 (IC_50_ = 30 nM). This aspect was reinforced in cellular assays by treating the B16-F10 cancer cell line with **18**. The increase in the rate of acetylated histone H3 by enhancing the concentration of **18** proved the HDAC inhibitory activity. The in vitro tubulin polymerization assay was then examined for **18**. The TPI property of **18** was thus confirmed with an IC_50_ value of 12.2 µM. This characteristic was also observed upon incubating **18** in B16-F10 cells, where **18** deeply disrupted the microtubule network. Compound **18** also inhibited cancer cell migration, especially at a concentration of 100 nM. Moreover, **18** interfered with the cell cycle by stopping mitosis at the G2/M phase, thereby inducing apoptosis. In vivo investigations of **18** using a mouse model (10 mg/kg dose of **18** (which was solubilized in a mixture of ethanol/polyoxyethylene castor oil/saline: 1.25/1.25/7.5) via the daily intraperitoneal route for two weeks) revealed a significant reduction in tumor growth (TGI = 70%). Furthermore, no notable changes in body weight were observed during the treatment period. Further safety assessments indicated that **18** did not have significant effects on major organ tissues, including the heart, liver, and kidney [112].

**Scheme 20:**
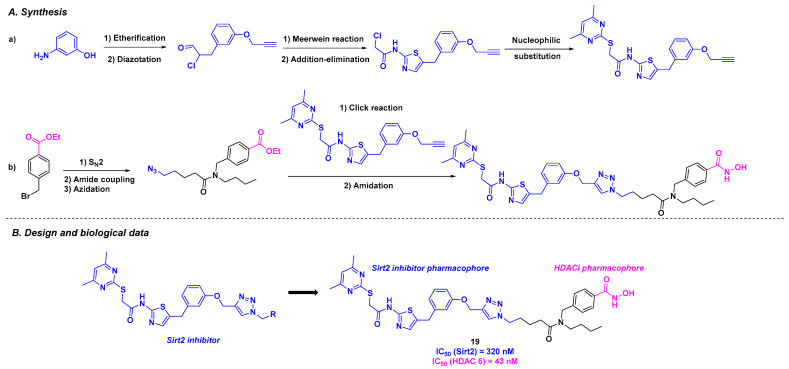
Dual Sirt2/HDAC 6 inhibitor developed by the Schiedel group [115].

In 2023, the Schiedel group designed an original dual molecule with two specific targets: tubulin deacetylases sirtuin 2 (Sirt2) and HDAC 6. As the disruption of the two enzyme activities could promote cancer processes [113], the team conceived a hybrid structure inspired by the Sirt2 and HDAC6 respective selective inhibitors (Scheme 20). Inspired by the Sirt2 inhibitors previously developed in the laboratory [114], Schiedel and his coworkers installed an aminothiazole core using a diazotation step followed by a Meerwein reaction. This motif was then attached to a hydroxamic acid skeleton, the HDACi pharmacophore, via copper-catalyzed Huisgen cycloaddition. The in vitro biological efficiency of these dual molecules was then analyzed. Among the synthesized compounds, **19** exhibited the highest selectivity and activity toward Sirt2 and HDAC6, with IC_50_ values of 320 nM and 43 nM, respectively. It should be highlighted that the co-crystallization of **19** and its analogs was achieved with the two respective targets, indicating a strong hydrophobic interaction between Sirt2 and the corresponding pharmacophore, as well as coordination of the hydroxamic acid with the Zn atom. In cellulo assays corroborated the in vitro results, with a high level of acetylated α-tubulin observed by incubating 20 µM of **19** in the PC-3M-cell line. To demonstrate the synergistic effect of hybrid molecule **19**, cell viability assays were performed on cancer cells sensitive to Sirt2 and HDAC 6 inhibition. **19** could decrease the viability of HGC27, W1, MCF-7, and PC-3M-luc cells, with EC_50_ values ranging from 12.9 to 30.1 µM [115].

## 10. 2-Methoxyestradiol Core

**Scheme 21:**
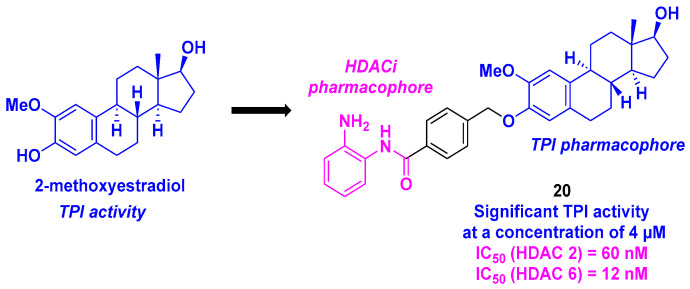
HDAC/tubulin dual inhibitor developed by Yao et al. [117].

2-Methoxyestradiol, a natural metabolite of estradiol, and its derivatives are known as remarkable tubulin polymerization inhibitors and disruptors of the microtubule skeleton [116]. In this context, Yao et al. described a dual-targeting inhibitor exhibiting a 2-methoxyestradiol scaffold as a TPI pharmacophore linked to a 2-aminobenzamide motif as an HDACi. After a thorough examination of the SAR for forty-seven synthesized hybrid molecules, **20** was identified as the compound exhibiting the best cytotoxicity against six cancer cell lines (MCF-7, MGC-803, HeLa, A549, HepG2, and U937), with IC_50_ values ranging from 0.371 to 4.840 µM. The inhibitory activities of **20** toward HDAC isoforms showed higher selectivity toward HDAC isoforms 2 and 6, with IC_50_ values of 60 and 120 nM, respectively (Scheme 21). In vitro immunofluorescence assays showed that compound **20** could disorganize the microtubule network. Moreover, incubating **20** at a concentration of 4 µM with purified porcine brain tubulin significantly inhibited tubulin polymerization. Similar to tubulin polymerization inhibitors, **20** efficiently halted the cell cycle at the G2/M phase, induced apoptosis via a change in mitochondrial membrane potential, increased the ROS rate, and upregulated pro-apoptotic proteins (cleaved forms of caspase 3, 7, 9, and PARP). In addition, **20** could also hamper the proliferation, migration, and invasion of tumor cells. All these antitumor activities were validated in an in vivo zebrafish xenograft tumor model (incubation for 1 h with different concentrations of **20** (1–4 µM) for 3 days) [117].

## 11. Millepachine Core

**Scheme 22:**
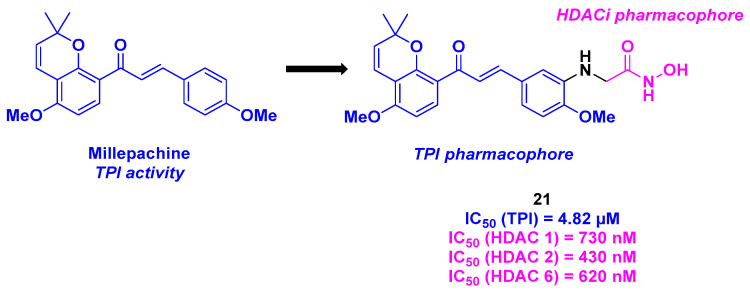
Dual HDAC/tubulin inhibitor developed by Yin, Kong and their colleagues [118].

More recently, Yin, Kong, and their colleagues developed a new family of dual HDAC/tubulin inhibitors featuring millepachine, a natural chalcone with TPI activity, branched to a hydroxamic acid structure as an HDACi (Scheme 22). After large-molecule-screening against five cancer cell lines (MDA-MB-231, A549, PC-3, U251, and MCF-7), compound **21** was determined to be the best molecule with the highest cytotoxicity, with a notable IC_50_ value of 16 nM for the human prostate cancer cell line PC-3. The HDAC inhibition activities were then inspected, showing that **21** possessed 67% inhibition of HDACs at a concentration of 1 µM. Furthermore, **21** exhibited pan-HDAC inhibitory activity toward HDAC 1, 2, and 6. The TPI properties of **21** were validated using purified tubulin protein, with an IC_50_ value of 4.82 µM. This result was confirmed by immunofluorescence assay, where intracellular microtubules were disassembled by incubation with **21**. The biological **21** action was then clarified using flow cytometry and Western blot. The authors demonstrated that **21** could interrupt the cell cycle at the G2/M phase by enhancing cleaved PARP and Caspase 3 levels and diminishing Bim and Bcl-2 levels. Compound **21** also inhibited the migration of tumor cells and was involved in the mitochondrial apoptosis process by reducing the mitochondrial membrane potential and increasing ROS levels. In addition, an anti-angiogenesis effect was detected with **21**, since a decrease in the HUVECs capillary-like tubular network was observed in the presence of **21**. Finally, all the in vitro antitumor aspects of **21** were ascertained in the in vivo PC-3 mice xenografts (intravenous injection every two days, for a three-week treatment), with a TGI of 90% at a **21** dosage of 20 mg/kg (N. B.: For the intravenous dose, **21** was dissolved in a mixture of DMSO/Tween 80/Saline (10/10/80)) [118].

## 12. Deoxypodophyllotoxin Derivatives

**Scheme 23:**
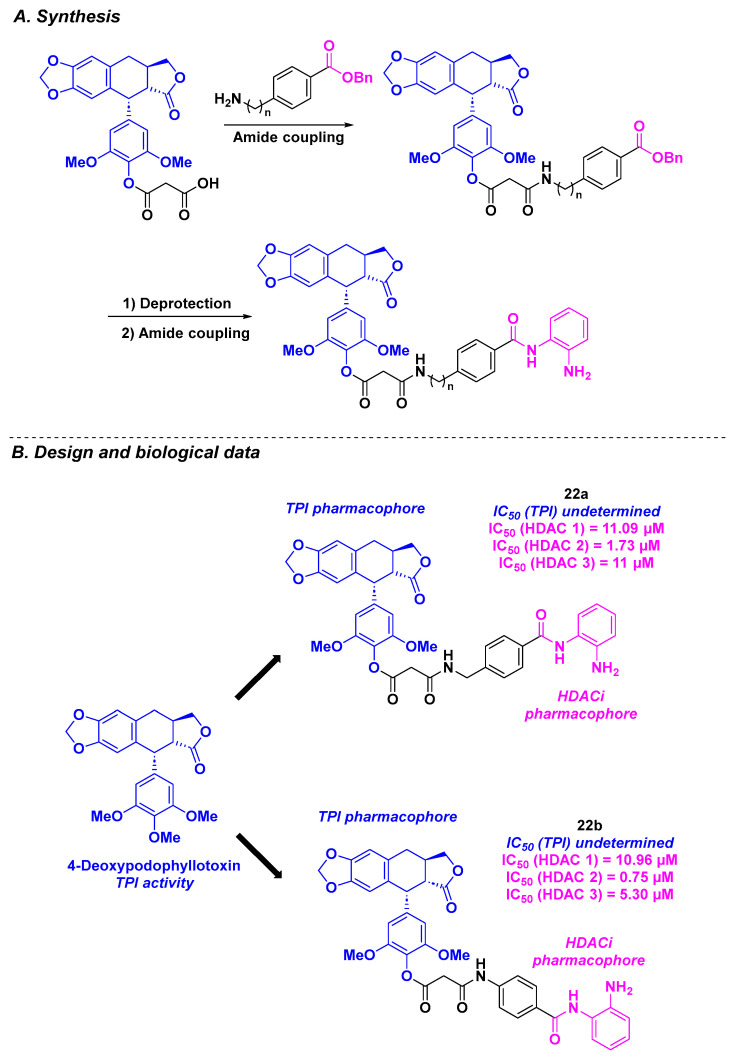
Dual HDAC/tubulin inhibitors elaborated by Chen, Li, Lu and coworkers [123].

In 2014, Chen, Li, Lu and coworkers proposed a dual HDAC/tubulin inhibitor, integrating a podophyllotoxin (PPT) analog platform. Based on the design of their topoisomerase II/HDAC hybrid inhibitors [119], the team elaborated an analogous approach for the construction of novel molecules. With the well-known TPI properties of deoxypodophyllotoxin (DPT) [120,121] combining with the HDACi activities of benzamides, a novel family of molecular hybrids could be generated (Scheme 23). The DPT core was synthesized from PPT in three steps under standard conditions [122]. The benzamide structure was then linked to the DPT group via amidation using HATU as the coupling agent. The in vitro HDAC inhibition of the established molecules disclosed a pan inhibitory activity for **22a** and **22b** toward HDAC 1, 2, and 3 (IC_50_ = 0.75–11.09 µM). By treating HCT-116 cells with dual HDAC/tubulin inhibitors at 40 or 80 nM for 24 h, the authors observed an accumulation of cells in the G2/M phase, indicating cell cycle blocking by the TPI effect. The cytotoxicity of the prepared structures validated the biological activities of **22a** and **22b** in A549 and HCT-116 cancer cell lines, with IC_50_ values ranging from 36 to 40 nM [123].

## 13. Paclitaxel Scaffold

**Scheme 24:**
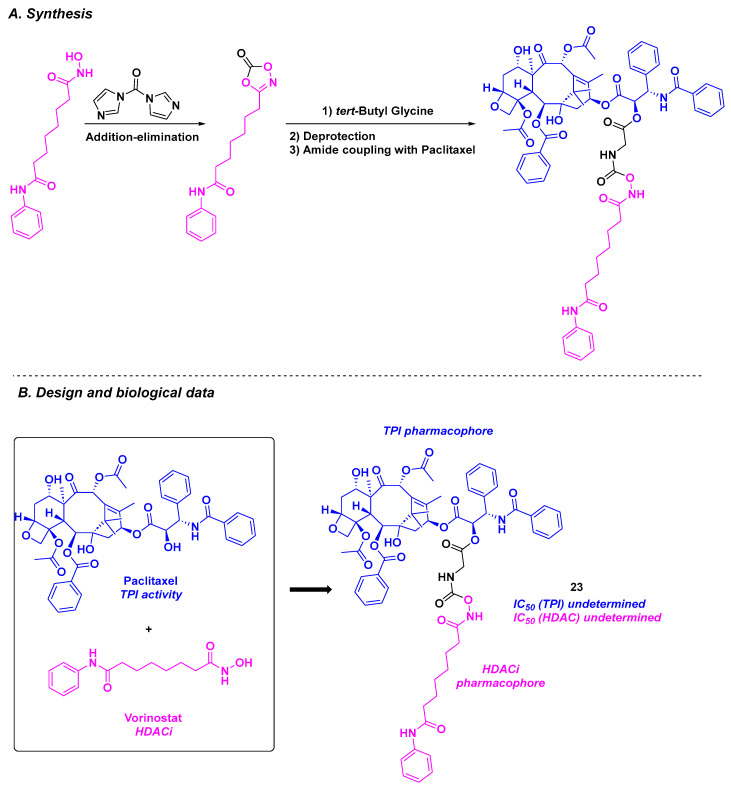
Dual HDAC/tubulin inhibitor conceived by the Chen and Lu groups [125].

Given the high efficiency of paclitaxel as an FDA-approved anticancer drug and its activity as a tubulin polymerization inhibitor [124], it would seem obvious to combine this molecule with an FDA-approved HDACi, such as vorinostat. This idea was proposed by Chen and Lu, who conjugated paclitaxel with vorinostat through a glycine or succinic acid linker [125] (Scheme 24). The cytotoxicity of the synthesized dual inhibitors was determined. Surprisingly, compound **23** bearing the glycine motif exhibited higher antiproliferative activity against the drug-resistant cancer cell line MCF-7/ADR compared to paclitaxel alone, with an IC_50_ value of 1384 nM. This promising result prompted the research teams to explore the synergistic effect of **23** at the cellular level. Compound **23** was able to increase G2/M cell cycle arrest compared to the standard influence of paclitaxel. Western blot analyses indicated that **23** induced hyperacetylation of tubulin in HCT-116, MCF-7, and MCF-7/ADR cells at a concentration of 10 nM.

## 14. Sulfonamide Scaffold

**Scheme 25:**
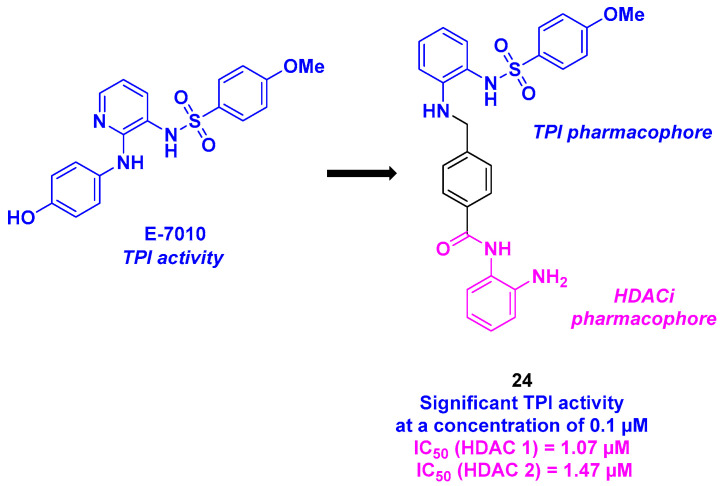
Molecular hybrids HDAC/tubulin inhibitor developed by Chen, Liou and coworkers [127].

The sulfonamide skeleton is present in the structure of E-7010, an orally active antitumor compound developed by the Eisai Company. This molecule provided promising biological results, namely, tubulin polymerization inhibition by binding to the colchicine-binding site [126]. Based on these findings, Chen, Liou, and coworkers envisioned the construction of hybrid agents, including a sulfonamide group for TPI activity and a benzamide function as an HDACi (Scheme 25). The connection between the two chemical entities was ensured using a stilbene link. As this motif is not a substrate for membrane-bound P-glycoprotein (P-gp), stilbene can bypass the P-gp-mediated multidrug resistance pathway. Based on this hypothesis, twenty-two dual molecules were prepared and biologically evaluated. Antiproliferative activity revealed that compound **24** was the most cytotoxic agent against KB cell lines, with a GI_50_ value of 12 nM. It should be highlighted that the same results were noticed in drug-resistant KB cancer cell lines. The HDAC isoform inhibition showed a strong selectivity of **24** for HDAC 1 and 2, with IC_50_ values of 1.07 and 1.47 µM, respectively. Mechanistic insights revealed that **24** could activate pro-apoptotic markers, such as activated PARP, γH2AX, and caspases 3, 8, and 9, in KB cell lines. In vitro complementary analyses using flow cytometry and fluorescence microscopy, the authors found that **24** blocked the cell cycle at the G2/M phase, with microtubule disassembly [127].

## 15. Indoline/Indole-Sulfonamide Scaffold

**Scheme 26:**
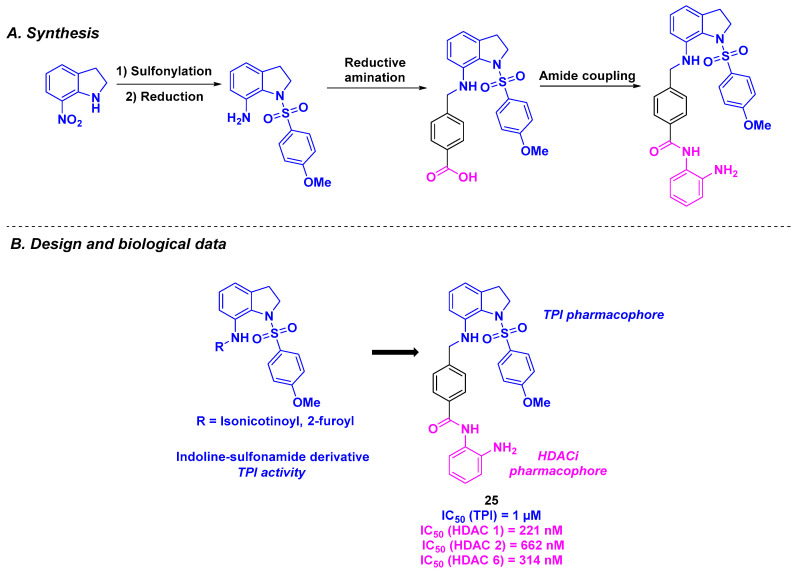
Hybrids HDAC/tubulin inhibitors developed by Chen, Liou and their colleagues [129].

With the promising biological properties of indoline-sulfonamide derivatives as tubulin inhibitors [128], Liou, Chang, and coworkers designed a hybrid dual molecule incorporating this scaffold and a benzamide group as an efficient HDAC pharmacophore (Scheme 26). The two functional motifs were associated with a benzyl linker installed through reductive amination under standard conditions. Twenty-one dual compounds were synthesized and subjected to biological assays. The antiproliferative activities were determined, with compound **25** being the most cytotoxic against KB, A549, and MKN45 cancer cell lines (IC_50_ = 49–79 nM). The cell-killing capacity of **25** was confirmed in oral epidermoid carcinoma drug-resistant cell lines (KBVIN10, KB-S15, and KB-7D), with IC_50_ values ranging from 44 to 65 nM. The TPI abilities of the prepared hybrid molecules were also measured. Compound **25** was identified as the best tubulin inhibitor, with an IC_50_ value of 1 µM. Further examinations showed that **25** had a better affinity for the colchicine site compared to colchicine at concentrations of 1 or 5 µM. HDAC isoform selectivity was detected for **25**, especially with a significant inhibition of HDAC 1, 2, and 6. Moreover, the HDACi efficiency of **25** was verified in A549 cells, with the accumulation of acetylated α-tubulin in the presence of **25** observed. Finally, the dual inhibitory feature of **25** was established in an in vivo A549 xenograft mouse model, with a remarkable reduction in size of the tumor (TGI = 62.9% with a **25** intraperitoneal daily dosage of 50 mg/kg, solubilized in DMSO/Cremophor/Dextrose: 5/5/90). Similar results were also found in the B-cell lymphoma xenograft tumor mouse model (daily intravenous dose of **25** (vehicled in DMSO/Cremophor/Saline: 10/20/70) at 50 mg/kg, 5 times a week) [129].

**Scheme 27:**
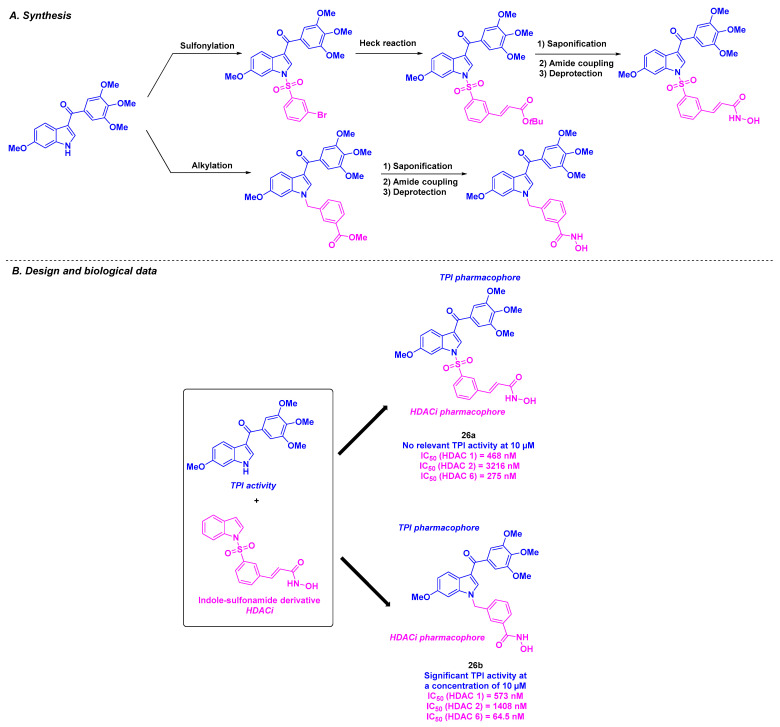
Hybrid HDAC/tubulin inhibitors conceived by Yang, Liou and coworkers [132].

The same group envisioned a library of dual-active compounds fusing a potent tubulin assembly inhibitor, currently in phase II clinical trials [130], with an HDACi bearing an indoline-sulfonamide structure [131] (Scheme 27). The construction of the hybrid molecules was based on a three-step synthesis featuring an alkylation step, Heck coupling, and amide condensation [132]. The developed structures demonstrated antitumor activity against A549, HCT-116, and PC-3 cancer cell lines (IC_50_ values from 179.26 to 484.57 nM) for compound **26a**. In contrast, despite its remarkable cytotoxic properties, the tubulin polymerization inhibitory property of **26a** remained less important than that of **26b**. In addition, **26a** and **26b** displayed high HDAC 6 specificity, with IC_50_ values of 275 and 64.5 nM, respectively. In vivo experiments on PC-3 xenograft mice models validated the efficacy of **26b**, with the suppression of tumor growth at daily oral doses of 100 and 200 mg/kg (TGI of 24.8% and 68.5%, respectively). Similar outcomes were obtained with multiple myeloma RPMI-8226 xenografts (TGI values of 35.8% and 58.2% for intraperitoneal daily doses of 50 and 100 mg/kg, respectively).

Emulating the previous research of the Liou team, Fu et al. deepened the investigation of the properties of chimeric indole-sulfonamide **26a** [133] (Scheme 27). Compound **26a** also exhibited significant antiproliferative activity against liquid tumor cell lines, such as HL-60, with an IC_50_ value of 42 nM. Molecular docking validated the dual targeting of **26a**, with a partial overlap with colchicine in the tubulin binding site and analogous interactions with vorinostat in HDAC active site. Further biological analyses indicated that this molecule was able to stop the mitosis process at the G2/M phase by significantly varying the G2/M transition protein rates. In addition, **26a** could induce cell apoptosis by upregulating pro-apoptotic proteins (cleaved caspases 3, 7, 8, 9, and PARP). Mouse xenograft models confirmed the antitumor effects of **26a**, with TGI values of 31.1 and 40.9% in PC-3 and HL-60 grafted mice respectively.

In parallel with the conventional molecular hybrid inhibitors connecting TPI and HDACi pharmacophores, some original dual molecules have recently been conceived based on their HDACi chemical structures.

## 16. Dual HDAC/Tubulin Inhibitors Inspired by the HDACis

Among the wide diversity of developed dual HDAC/tubulin inhibitors, those the originating from HDACis constitute an important molecule family. In addition to their main function on histones, HDACs could also react with non-histone proteins, such as α-tubulin. For example, HDAC 6 could use tubulin as a substrate, thus regulating the stability of the microtubule network [134,135,136]. Therefore, the development of HDACis with dual HDAC/tubulin activity has emerged. In this frame, medicinal chemists mainly based their compound designs on HDACis developed in the literature. The tubulin inhibitory activity of the synthesized molecules was systematically studied using diverse biological assays.

### 16.1. Pyrrolo [2,3-d]pyrimidine Skeleton

**Scheme 28:**
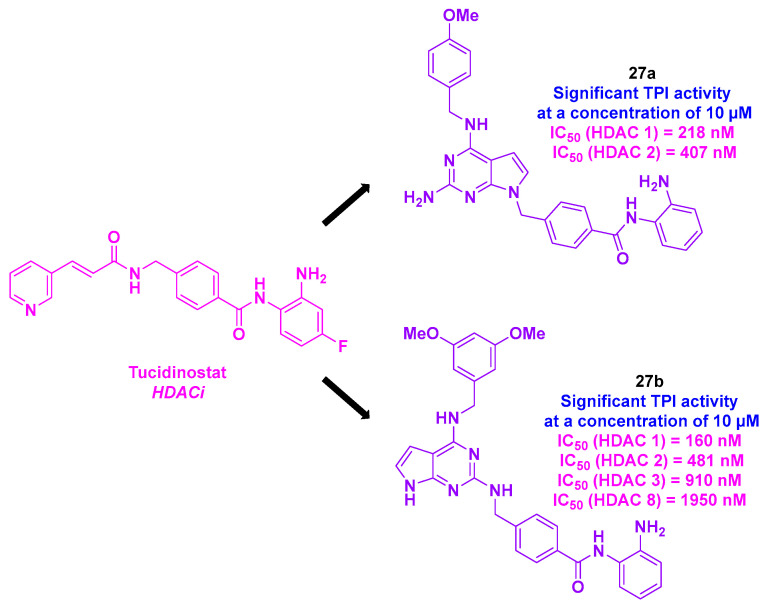
Dual HDAC/tubulin inhibitor designed by Lee, Liou and their colleagues [139].

Emulating the structure of tucidinostat and their recent advances in the development of HDACis [137], Lee, Liou, and their colleagues envisaged at the first glance an ameliorated version of their HDACis (Scheme 28). In this prospect, the team elaborated a rigid and bulky pyrrolo [2,3-*d*]pyrimidine structure at the cap part of the HDACis, improving the anticancer properties of the molecules by efficiently blocking the active site entrance [137]. As some reported HDACis also target tubulin [138], the group considered their newly designed HDACis as potential dual hybrid HDAC/tubulin inhibitors. The in vitro cytotoxicities of these molecules were then studied. Among the nine synthesized compounds, **27a** and **27b** exhibited the highest antiproliferative activities against eight solid tumor cell lines (MDA-MB-231, MDA-MB-468, HeLa, DLD-1, HCT-116, H661, H1299, and A549), with IC_50_ values ranging from 50 to 1150 nM. It should be noteworthy that these cytotoxicity properties were higher than those of tucidinostat. Likewise, **27a** and **27b** confirmed their antitumor effects in leukemia cell lines (HH, HuT78, HL60, and KG-1), with IC_50_ values ranging from 80 to 150 nM. Moreover, **27a** exhibited significant cell-killing efficacy in the multidrug-resistant cell line MES-SA/Dx5, with an IC_50_ value of 9.54 µM, three times higher than that of vinorelbine.

The analysis of HDAC isoform inhibitory activities highlighted that **27a** had a strong selectivity for HDAC 1 and 2, whereas **27b** showed a wider pan-HDAC inhibitory efficiency for HDAC 1, 2, 3, and 8. Mechanistic studies using flow cytometry suggested that **27a** and **27b** could promote apoptosis in tumor cells by inducing the production of active forms of caspases-3 and -9 and reducing the formation of anti-apoptotic agents MCL-1 and Bcl-xL. The TPI of **27a** and **27b** was also scrutinized using an in vitro tubulin polymerization assay. By incubating tubulin proteins with 10 µM of **27a** and **27b**, the authors noted that these compounds were able to jeopardize the microtubule assembly. It should be noted that no in vitro inhibition was found for HDAC 6 for **27a** and **27b**, implying an independent TPI pathway for the two dual compounds [139].

### 16.2. Benzamide Scaffold

**Scheme 29:**
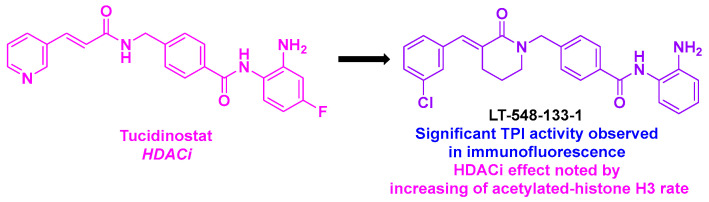
Dual HDAC/tubulin inhibitor **LT-548-133-1** reported by He, Chen and coworkers [140].

In 2021, He, Chen, and coworkers described **LT-548-133-1**, a tucidinostat analog possessing equivalent HDAC inhibitory activity [140] (Scheme 29). When **LT-548-133-1** was incubated with MCF-7 cells for 48 h, an IC_50_ value of 2.1 mM was obtained. Further investigations indicated that **LT-548-133-1** could enhance the rate of acetylated histone H3, probably due to its HDAC inhibitory property. Moreover, **LT-548-133-1** promoted cell cycle arrest at the G2/M phase, contrary to tucidinostat, which induced G0/G1 cell mitosis arrest. This result indicated that **LT-548-133-1** might follow a different mechanism of action compared to tucidinostat. Complementary western blot analyses indicated that G2/M cell cycle arrest was induced by an increase in CyclinB1 protein expression. In addition, **LT-548-133-1** could lead to abnormal cell mitosis and apoptosis in MCF-7 cell lines. Based on these effects on the mitosis process, the authors suggested that **LT-548-133-1** could interfere with the microtubule network. Putting **LT-548-133-1** with MCF-7 cells revealed by immunofluorescence the destructuration of the microtubules, proving the TPI effect of **LT-548-133-1**.

### 16.3. Quinazoline Scaffold

By scrutinizing the biological pathway of their previously developed **SKLB-23bb** [141], an HDAC 6 selective inhibitor, the Chen group revealed that this molecule could have a dual-targeting activity [142].

**Scheme 30:**
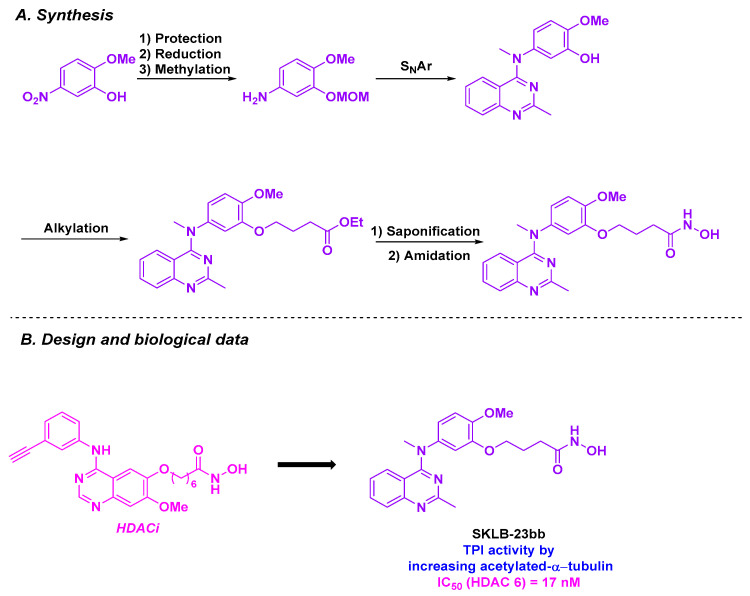
Dual HDAC/tubulin inhibitor **SKLB-23bb** developed by the Chen group [142].

**SKLB-23bb** contains a 2-methylquinazoline core, a known key bioactive skeleton [53,143], linked to a hydroxamic acid group through an alkyl linker (Scheme 30). **SKLB-23bb** was obtained through a S_N_Ar reaction involving a highly functionalized aniline, followed by amide condensation [141]. This molecule exhibited high cytotoxic potential, with nanomolar-range IC_50_ values (36.68 to 116.56 nM) across a wide panel of liquid and solid cancer cell lines. In addition to its HDAC 6 inhibitory property, **SKLB-23bb** also acts via another biological pathway to hamper cancer cells. The cytotoxic effects of **SKLB-23bb** were similar in HDAC 6 knockout tumor cell lines compared to wild-type cells. To clarify the exact mechanism of action of this molecule, **SKLB-23bb** was incubated with fluorescent-stained A2780s cell line. Major morphological modifications in the microtubule network were noted, and microtubule dysfunction was similarly observed upon addition of colchicine to A2780s cells. This result could suggest that this molecule could also bind to the colchicine site of tubulin to block the polymerization process. The validation of this hypothesis was observed in the EBI competition assay. Complementary TPI tests demonstrated that **SKLB-23bb** could inhibit tubulin polymerization at a concentration of 10 µM. As a result of this biological characteristic, **SKLB-23bb** could stop the cell cycle at the G2/M phase and trigger apoptosis by promoting the upregulation of pro-apoptotic proteins, such as Bax.

The effectiveness of **SKLB-23bb** was preserved in vivo in xenograft mice imitating the B-lymphoma model, with a tumor growth inhibition (58.22% tumor-inhibitory rate) at an oral dose of 40 mg/kg three times a week. Analogous results were obtained with solid tumor models (HCT-116 and A2780 xenografts, with an oral dose of 6 or 12.5 mg/kg, thrice a week).

### 16.4. Imidazolyl Motif

**Scheme 31:**
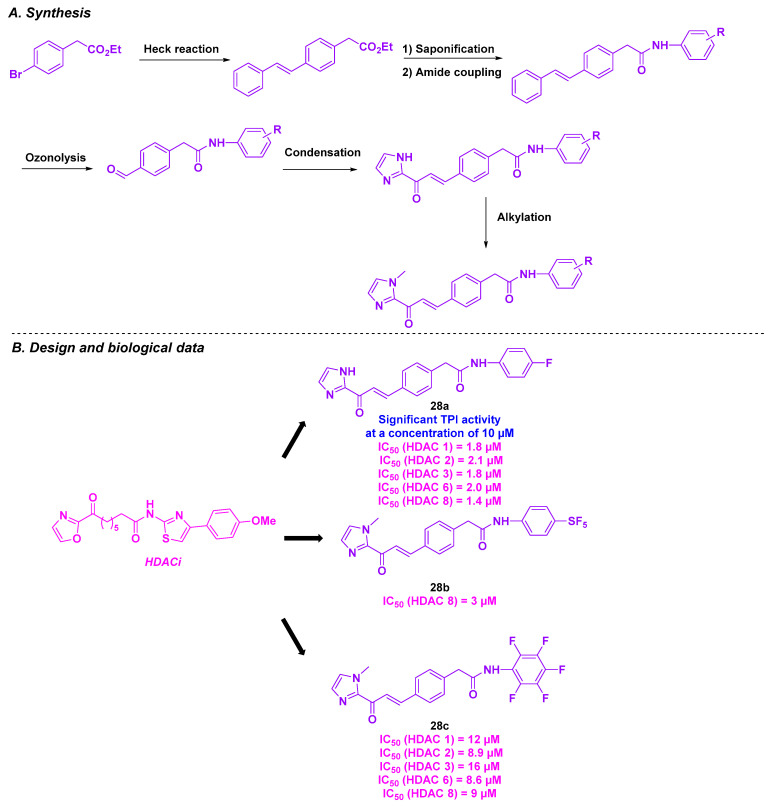
Dual HDAC/tubulin inhibitor conceived by Taylor, Tillekeratne et al. [148].

In 2021, Taylor, Tillekeratne et al. developed a novel class of heterocyclic compounds with a dual action on HDACs and tubulin. Inspired by imidazole derivatives and FDA-approved hydroxamic acids as HDACi [144,145,146], the group introduced an imidazolyl skeleton with potential HDAC inhibitory activity. To strengthen the dual property of the newly conceived compounds, a chalcone scaffold was added to the molecule’s structure to impart antimitotic activity (Scheme 31) [147]. The synthesis of these dual inhibitors was achieved via a multi-step synthesis, including a Heck cross-coupling reaction, olefin oxidative cleavage, and aldol condensation as key steps. A preliminary biology study on the HCT-116 cell line was conducted using the prepared compounds, confirming their cytotoxicity in the micromolar range (IC_50_ = 5.14–6.95 µM). The inhibitory effects of the HDAC isoforms were then evaluated. The in vitro results highlighted that compounds **28a** and **28c** had pan-HDAC inhibitory character toward HDAC 1, 2, 3, 6, and 8. In contrast, molecule **28b** was a selective HDACi for HDAC 8. A similar analysis was carried out by incubating the synthesized compounds with HCT-116 cells. Unexpectedly, no significant variation in histone or tubulin acetylation was observed, implying that these molecules did not mainly target HDACs. Despite these outcomes, complementary assays were performed to evaluate the cytotoxicity of these compounds against a wide range of cancer cell lines. The compounds demonstrated their effectiveness in HeLa and NCI-60 cancer cell lines with micromolar GI_50_ values. Examination of the antimitotic activity of **28a** in HeLa cells revealed that **28a** was able to arrest the mitosis process, by destabilizing microtubules. Molecular docking studies have indicated a potential interaction between **28a** and tubulin at the colchicine-binding site [148].

## 17. Conclusions

The rise of dual-targeting drugs during the last decades represents a promising alternative to conventional chemotherapeutics in cancer treatment. By combining two pharmacophores into a single anticancer agent, medicinal chemists aim to target “two birds in one stone” thereby overcoming the limitations of traditional chemotherapy, such as excessive toxicity, reduction of immunity, and development of drug resistance.

As highlighted in this review, tubulin and HDACs are highly effective and complementary targets for the development of dual-active agents. The remarkable efficacy of the described dual molecules is evident from their outstanding in vitro antiproliferative activities, with nanomolar IC_50_ values in various human cancer cell lines. These compounds exhibited pan-HDAC inhibitory properties, notably against HDAC 1, 2, and 6 (IC_50_ in satisfactory nanomolar ranges), and effectively inhibited tubulin polymerization (favorable IC_50_ micromolar values), which further led to the arrest of the cell cycle at the G2 phase. Some of the HDAC/tubulin inhibitors, such as CA-4 derivatives, also display potent anti-angiogenic properties, contributing to their antitumor characteristics. In addition to their synergistic effects on HDACs and tubulin, these inhibitors showed minimal toxicity at both the cellular and in vivo levels, highlighting their potential for therapeutic application (Table 1).

However, despite their promising preclinical results, no dual HDAC/tubulin inhibitors have entered clinical phase trials yet, as the pharmacokinetics of the most advanced hit molecules are still under investigation. Furthermore, complementary studies should be conducted on dual compounds. For instance, as reported in Table 1, only three agents presented good water solubility (up to 138 µg/mL). Physicochemical data for the other compounds need to be explored. In vivo studies should also be performed for some derivatives after in vitro studies encouraging outcomes.

Hence, the conception of dual-targeting drug candidates remains a challenge in cancer therapy. The elaboration of such molecules would require substantial efforts to synthesize lead compounds, along with thorough pharmacomodulation to meet pharmacokinetics and toxicity criteria.

## Figures and Tables

**Table 1:** Summary table of HDAC-tubulin dual-targeting inhibitors.

Compounds		IC_50_ for Cancer Cells	IC_50_ for Normal Cells	IC_50_ (TPI)	IC_50_ (HDAC)	In Vivo Results	Water Solubility	References
CA-4 derivatives	Chalcone derivative **1a**–**c**	Up to 550 nM for A549 cells	n. d.	Up to 5.0 µM	4.6–4910 nM (HDACs 1, 6 and 8)	n. d.	n. d.	[82]
	Chalcone derivative **2a**–**c**	From 1.62 to 2.21 µM against MCF-7 and Hep G2 cells	n. d.	Up to 2.32 µM	4.6–3880 nM (HDACs 1, 6 and 8)	n. d.	n. d.	[83]
	Stilbene derivative **3a**–**b**	From 0.4 to 5.1 nM for A549, K562, K562R, PC3, U87-MG, MCF7, BXPC3, MiaPaca2, and HT29 cells	7 µM	Up to 1.6 µM	30–340 nM (HDAC 8)	n. d.	n. d.	[87]
	Stilbene derivative **4a**–**b**	ca. 0.65 nM for NCI-N87, K562, K562R, MiaPaca2, SKOV3, A549, MCF7, MDA MB231 and HT-29 cells	n. d.	Up to 20 nM	150–280 nM (HDAC 8)	Significant tumor decrease for 0.25–0.50 mg/kg doses of **4a**	From 68.7 to 65 µg/mL	[88]
	Stilbene derivative **5**	From 16 to 305 nM for MGC-803, MCF-7, U937, A549, HepG2 and HeLa	195–556 nM	1.2 µM	50–370 nM (HDACs 3–9)	Significant tumor decrease at a concentration of 100 nM	n. d.	[89]
	Stilbene derivative **6**	From 3 to 36 nM for K562, MCF-7, MDA-MB-231, A549, B16F10 and A2780 cells	230 nM	3.84 µM	403–591 nM (HDACs 1, 2 and 6)	TGI = 73.12% for daily 20 mg/kg dose	n. d.	[90]
	Amine derivative **SY-65**	From 39 to 254 nM for MGC-803, HGC-27, SGC-7901, HCT-116 and KYSE450 cells	n. d.	3.64 µM	525–5270 nM (HDACs 1–3)	Inhibition of the tumor growth at a 30 mg/kg dose	n. d.	[91]
	Oxazole derivatives **7a**–**c**	From 0.11 to 14.2 µM for 518A2, 518A2, HT-29, DLD-1, HCT-116, KB-V1^Vbl^ and MCF-7^Topo^ cells	n. d.	TPI activity at 10 µM of **7a**	Down to 320–13,800 nM (HDAC 1 and 6)	Low toxicity at up to 200 mg/kg of **7a** in nude mice model	n. d.	[92]
	1,4-Diarylazetidin-2-one derivatives **8a**–**b**	From 16 to 56 nM for BE-(2)-C, A549, U87MG and HCT-116 cells	n. d.	5.4 µM for **8b**	Down to 155–2405 nM (HDACs 1, 6 and 8)	TGI = 67% for 25 mg/kg dose of **8b** every 3 days	102.2 µg/mL	[94]
	Arylpyridine derivative **9**	From 17 to 90 nM for BE-(2)–C, A549, U87MG and HCT-116 cells	170 nM	2.4 µM	117–5839 nM (HDACs 1, 6 and 8)	TGI = 61% for 50 mg/kg dose every 3 days	138 µg/mL	[95]
	Arylpyridine derivative **10a**–**b**	For **10a**: 3.52 µM for UO-31 cells and 5.50 µM for T47D cells	n. d.	Up to 3.73 µM	57–84 nM (HDACs 1 and 6)	n. d.	n. d.	[97]
Colchicine derivatives	**11a**–**b**	For **11b**: from 0.242 to 4.672 µM for A431, A549, HCT-116, MCF-7 and PC-3 cells	n. d.	n. d.	440–3430 nM (HDACs 1, 3 and 6)	n. d.	n. d.	[98]
	**12a**–**b**	From 2 to 106 nM for A549, HCT-116, SW620, Hep3B, HeoG2, MHCC97H, SNU-5, SNU-16, MKN-45, PANC-1 and SJSA-1 cells	n. d.	TPI activity at 10 µM	0.19–12.5 µM (HDACs 1–3)	n. d.	n. d.	[99]
Aminobenzamide derivative	**13**	From 26 to 30 nM for HepG2, HCT-116, MDA-MB-231, H22 and MCF-7 cells	134 nM	4.06 µM	115–242 nM (HDACs 1–6)	Reduction of the tumor weights by 82% for daily 20 mg/kg dose	n. d.	[101]
Amide derivatives	**14a**–**b**	From 0.65 to 0.92 µM for HepG2	From 9.62 and 11.09 µM for normal liver HL-7022 cells	270 nM for **14a**	For **14a**: 47 nM (HDAC 1) and 86 nM (HDAC 2)	n. d.	n. d.	[102]
Quinolone derivative	**15**	From 0.3 to 4.9 µM for A549, HepG2, MCF-7, PC-3 and HeLa cells	>100 µM	1.79 µM	21–41 nM (HDACs 1, 2 and 6)	n. d.	n. d.	[104]
Benzofuran derivatives	**16a**–**d**	From 0.4 to 23.5 nM for HeLa, MDA-MB-231, A549, HT-29, and MCF-7 cells	n. d.	From 420 to 580 nM	From 6 to >30 µM (HDACs 1, 6 and 10)	n. d.	n. d.	[106]
2-Benzylideneindanone derivatives	**17a**–**b**	From 0.36 to 49.67 µM for MCF-7, MD-AMB-231 and K562 cells	From 47.23 to 100.32 µM for Vero cells	TPI activity at 5 µM	Residual activity for HDAC 6 from 0.03 to 0.09	Low toxicity at up to 1000 mg/kg of **17a**	1.76 µg/mL for **17b**	[110]
Aminothiazole derivatives	**18**	From 30 to 140 nM for HCT-116, B16-F10, Jurkat and A549 cells	n. d.	12.2 µM	30 nM (HDAC 3)	TGI = 70% for daily 10 mg/kg dose	n. d.	[112]
	**19**	EC_50_ from 12.9 to 30.1 µM for HGC27, W1, MCF-7, and PC-3M-luc cells	n. d.	320 nM (for Sirt2)	43 nM (HDAC 6)	n. d.	n. d.	[115]
2-Methoxyestradiol derivative	**20**	From 0.371 to 4.840 µM for MCF-7, MGC-803, HeLa, A549, HepG2 and U937 cells	8.14–10.89 µM	TPI activity at 4 µM	60 nM (HDAC 2) and 12 nM (HDAC 6)	Antitumor activity 1 to 4 µM 3-day incubation	n. d.	[117]
Millepachine derivative	**21**	From 13 nM to 340 nM for MDA-MB-231, A549, PC-3, U251 and MCF-7 cells	n. d.	4.82 µM	430–730 nM (HDACs 1, 2 and 6)	TGI = 90% for 20 mg/kg dose every two days	n. d.	[118]
Deoxypodophyllotoxin derivatives	**22a**–**b**	From 36 to 40 nM for A549 and HCT-116 cells	n. d.	n. d.	0.75–11.09 µM (HDACs 1–3)	n. d.	n. d.	[123]
Paclitaxel derivative	**23**	1384 nM for MCF-7/ADR cells	n. d.	n. d.	n. d.	n. d.	n. d.	[125]
Sulfonamide derivative	**24**	GI_50_ from 12 to 22 nM for KB and drug-resistant KB cells	n. d.	TPI activity at 0.1 µM	1.07 µM (HDAC 1) and 1.47 µM (HDAC 2)	n. d.	n. d.	[127]
Indoline/indole-sulfonamide derivative	**25**	From 44 to 79 nM for KB, A549, MKN45, KBVIN10, KB-S15 and KB-7D cells	n. d.	1 µM	221–662 nM (HDACs 1, 2 and 6)	TGI = 62.9% for daily 25 mg/kg dose	n. d.	[129]
	**26a**–**b**	From 179.26 to 6684.85 nM for A549, HCT-116 and PC-3 cells	n. d.	TPI activity at 10 µM for **26b**	64.5–3216 nM (HDACs 1, 2 and 6)	TGI = 68.5% for daily 200 mg/kg dose of **26b**	n. d.	[132]
Pyrrolo [2,3-d]pyrimidine derivative	**27a**–**b**	From 50 to 1150 nM for MDA-MB-231, MDA-MB-468, HeLa, DLD-1, HCT-116, H661, H1299 and A549 cells	n. d.	TPI activity at 10 µM	160–1950 nM (HDACs 1, 2, 3 and 8)	n. d.	n. d.	[139]
Benzamide derivative	**LT-548-133-1**	2.1 mM for MCF-7 cells	n. d.	TPI activity in immunofluorescence	HDACi effect with increase of acetylated-histone H3 rate	n. d.	n. d.	[140]
Quinazoline derivative	**SKLB-23bb**	From 36.68 to 116.56 nM for 23 cancer cell lines	n. d.	TPI activity with acetylated α-tubulin	17 nM (HDAC 6)	TGI = 58.22% for a 40 mg/kg dose three times a week	n. d.	[142]
Imidazolyl derivative	**28a**–**c**	5.14–6.95 µM for HCT 116 cells, GI_50_ with micromolar values for a wide panel of cancer cells	n. d.	TPI activity at 10 µM for **28a**	1.4–12 µM (HDACs 1, 2, 3, 6 and 8)	n. d.	n. d.	[148]

n. d.: not determined.
